# The HIV-1 accessory proteins Nef and Vpu downregulate total and cell surface CD28 in CD4^+^ T cells

**DOI:** 10.1186/s12977-018-0388-3

**Published:** 2018-01-12

**Authors:** Emily N. Pawlak, Brennan S. Dirk, Rajesh Abraham Jacob, Aaron L. Johnson, Jimmy D. Dikeakos

**Affiliations:** Department of Microbiology and Immunology, Schulich School of Medicine and Dentistry, University of Western Ontario, Dental Sciences Building, Room 3007J, London, ON N6A 5C1 Canada

**Keywords:** HIV-1 Nef, HIV-1 Vpu, CD28 downregulation, T cell activation

## Abstract

**Background:**

The HIV-1 accessory proteins Nef and Vpu alter cell surface levels of multiple host proteins to modify the immune response and increase viral persistence. Nef and Vpu can downregulate cell surface levels of the co-stimulatory molecule CD28, however the mechanism of this function has not been completely elucidated.

**Results:**

Here, we provide evidence that Nef and Vpu decrease cell surface and total cellular levels of CD28. Moreover, using inhibitors we implicate the cellular degradation machinery in the downregulation of CD28. We shed light on the mechanisms of CD28 downregulation by implicating the Nef LL_165_ and DD_175_ motifs in decreasing cell surface CD28 and Nef DD_175_ in decreasing total cellular CD28. Moreover, the Vpu LV_64_ and S_52/56_ motifs were required for cell surface CD28 downregulation, while, unlike for CD4 downregulation, Vpu W_22_ was dispensable. The Vpu S_52/56_ motif was also critical for Vpu-mediated decreases in total CD28 protein level. Finally, the ability of Vpu to downregulate CD28 is conserved between multiple group M Vpu proteins and infection with viruses encoding or lacking Nef and Vpu have differential effects on activation upon stimulation.

**Conclusions:**

We report that Nef and Vpu downregulate cell surface and total cellular CD28 levels. We identified inhibitors and mutations within Nef and Vpu that disrupt downregulation, shedding light on the mechanisms utilized to downregulate CD28. The conservation and redundancy between the abilities of two HIV-1 proteins to downregulate CD28 highlight the importance of this function, which may contribute to the development of latently infected cells.

**Electronic supplementary material:**

The online version of this article (10.1186/s12977-018-0388-3) contains supplementary material, which is available to authorized users.

## Background

Adaptive immune responses are primarily driven by B and T lymphocytes, which mediate humoral and cell-mediated immunity (reviewed in [1]). Thymus derived lymphocytes, or T cells, can be classified as CD8^+^ cytotoxic T-lymphocytes (CTLs) or CD4^+^ T helper cells, and these cells play key roles in anti-viral responses. Naïve T cells require signaling from antigen presenting cells (APCs) to become competent effector cells. According to the two-signal model of T cell activation, APCs provide two signals to enable these cells to become productive effector cells. The first signal is established during MHC-restricted binding of the T cell receptor (TCR): antigen complex, while signal 2 consists of co-stimulatory signaling (reviewed in [2, 3]). A lack of co-stimulatory signaling results in cells becoming unresponsive or anergic. Key co-stimulatory signaling is provided by binding of CD28, a transmembrane receptor expressed on the surface of T cells, to B7.1/B7.2 (CD80/CD86) on the surface of APCs. Indeed, CD28 receptor ligation initiates downstream signaling, which in conjunction with TCR signaling, results in cell activation and proliferation essential for mounting an immune response [4, 5].

The importance of the TCR and co-stimulatory signaling to the development of an effective immune response makes them prime targets of intracellular pathogens. Indeed, many viruses have evolved the capabilities to intricately modulate these T cell activation cues to optimize their replication and persistence. For instance, lymphotropic viruses, including the Human Immunodeficiency Virus Type 1 (HIV-1), Human T-lymphotropic virus (HTLV), and Epstein–Barr virus (EBV), inhibit T cell activation by downregulating components of the TCR and TCR-associated kinases essential for TCR signaling [6–11], as well as re-organizing the TCR and immunological synapse [12]. Moreover, viruses inhibit signaling downstream of co-stimulatory receptors, or alter the levels of cell surface co-stimulatory or inhibitory molecules [13–16]. Ultimately, virus-induced changes in T cell activation can result in immune evasion, enhanced replication, and increased persistence. The importance of viral alterations to immune cell activation are evidenced by the specific HIV-1 proteins that play key roles in T cell activation.

HIV-1 encodes four accessory proteins that lack any known enzymatic or structural functions: Vif, Vpr, Nef and Vpu (reviewed in [17–19]). Collectively, these viral proteins play critical roles in increasing infectivity, persistence and pathogenesis. While Nef and Vpu are arguably the most extensively studied accessory proteins, their roles in T cell activation are not fully understood. The ability of Nef and Vpu to downregulate various host cell receptors is a key function mediating their effects. Indeed, both Nef and Vpu downregulated multiple receptors in a screen to identify cell surface proteins that are altered by these viral proteins [20]. Specifically, Nef and Vpu facilitate degradation or intracellular sequestration of host receptors, including restriction factors and immune cell receptors, to thwart their cell surface expression [17]. By hijacking membrane trafficking proteins, such as the adaptor proteins adaptor protein 1 (AP-1) or adaptor protein 2 (AP-2), Nef and Vpu connect multiple cellular receptors to additional cellular trafficking machinery which alters the receptors’ subcellular localization. For example, Nef hijacks AP-1 to facilitate the endocytosis and sequestration of major histocompatibility complex type I (MHC-I) molecules [18, 21–23], limiting recognition of infected cells by the immune system [24]. In parallel, the ability of Nef to hijack AP-2 results in the endocytosis and degradation of CD4, which limits superinfection and antibody-dependent cell-mediated cytotoxicity [25–32]. Similarly, Vpu hijacks bone marrow stromal antigen 2 (BST-2) trafficking by associating with adaptor proteins to facilitate its sequestration and degradation [33–37]. Vpu also enables the degradation of CD4 by targeting newly synthesized CD4 to the endoplasmic-reticulum-associated protein degradation (ERAD) pathway [38]. Interestingly, Nef expression leads to endocytosis of CD28 from the cell surface, in a manner dependent on both AP-1 and AP-2 [39, 40]. However, the fate of CD28 after Nef-mediated endocytosis remains poorly understood and the effects of Vpu on CD28 are unexplored.

In this article, we report that Nef and Vpu decrease cell surface and total cellular CD28 levels within infected CD4^+^ T cells. Moreover, we can inhibit the observed decreases in total CD28 using inhibitors of the cellular degradation machinery, consistent with Nef and Vpu facilitating trafficking of CD28 to an acidic compartment. Additionally, a mutant Nef protein associated with impaired binding to the vacuolar ATPase was compromised in its ability to reduce cell surface and total CD28 levels, while an non-phosphorylatable Vpu protein that is unable to recruit the protein degradation machinery was impaired in its ability to reduce cell surface and total CD28 levels. Finally, the ability of Vpu to downregulate CD28 is not limited to the lab adapted HIV-1 strain NL4.3 Vpu, and infection of cells with viruses encoding Nef or Vpu have differential effects on activation upon stimulation of CD3 and CD28.

## Results

### The HIV-1 accessory proteins Nef and Vpu downregulate cell surface and total CD28 protein levels

**Fig. 1:**
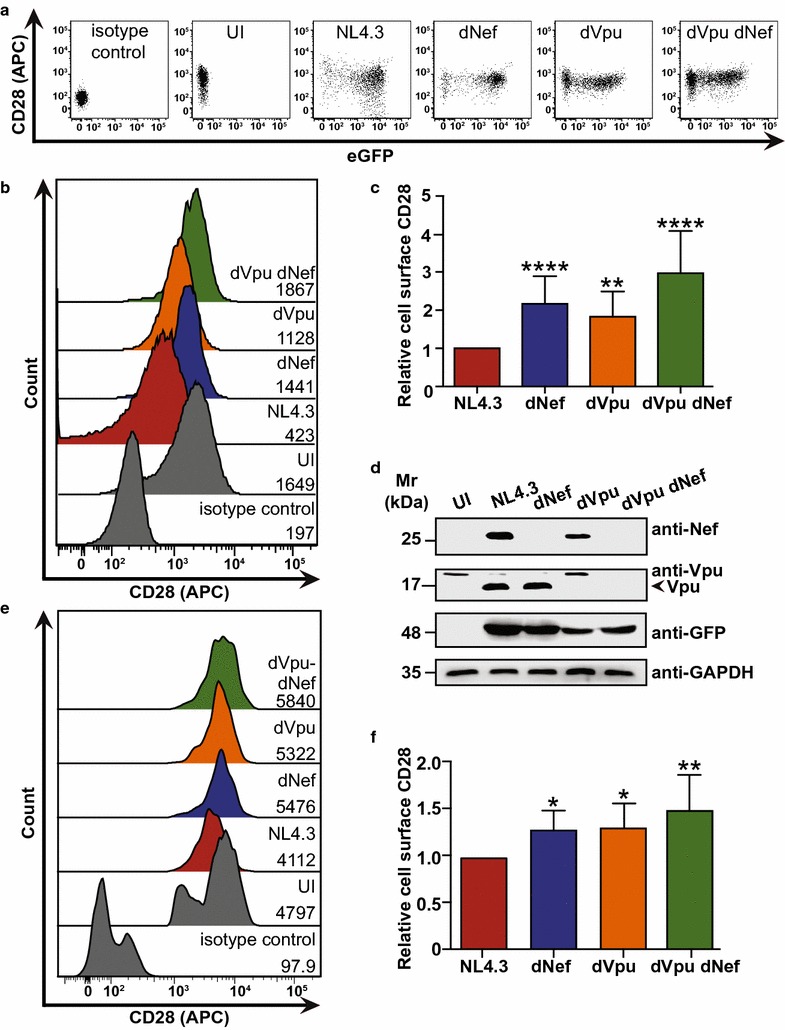
HIV-1 Nef and Vpu downregulate cell surface CD28 protein levels. CD4^+^ Sup-T1 and primary CD4^+^ T cells were infected with either VSV-G pseudotyped or replication competent NL4.3, respectively. Viruses encoded Nef and Vpu (NL4.3, red), lacked Nef (dNef, blue) or Vpu (dVpu, orange), or lacked both Nef and Vpu (dVpu dNef, green). Infected cells were stained for CD28 and analyzed by flow cytometry. Live infected Sup-T1 cells were analyzed by gating on Zombie Red^TM−^ and GFP^+^ cells, and infected primary CD4^+^ T cells were analyzed by gating on p24^+^ cells. **a** Representative dot plots illustrating cell surface CD28 (APC) and infection (GFP^+^) of live (Zombie Red^TM−^) Sup-T1 cells. **b** Representative histograms illustrating cell surface levels of CD28 or the appropriate isotype control on Sup-T1 cells after gating on live (Zombie Red^TM−^) and infected (GFP^+^) cells. CD28 (APC) geometric mean fluorescence intensities (MFI) are indicated. **c** Summary of the relative mean (± SE) cell surface CD28 levels on infected (GFP^+^) Sup-T1 cells based on MFIs (n = 17). **d** Western blot illustrating expression of Nef and Vpu in infected Sup-T1 cells. **e** Representative histograms illustrating cell surface levels of CD28 or the appropriate isotype control on uninfected (UI) or infected (p24^+^) CD4^+^ PBMCs. MFIs are indicated. **f** Summary of the relative mean (± SE) cell surface CD28 levels on infected CD4^+^ T cells based on MFIs obtained from infection of CD4^+^ T cells from two healthy donors (n ≥ 3). (UI: uninfected; SE: standard error; Mr: molecular weight; GAPDH: glyceraldehyde 3-phosphate dehydrogenase; GFP: green fluorescent protein; MFI: geometric mean fluorescent intensity; SE: standard error; *p ≤ 0.05; **p ≤ 0.01; ****p ≤ 0.0001)

The co-stimulatory molecule CD28 is essential for immune cell activation and proliferation of naïve and memory T cells [2]. To investigate the effects of the HIV-1 accessory proteins Nef and Vpu on endogenous CD28 levels, CD4^+^ Sup-T1 T cells were infected with Gag-Pol truncated, VSV-G pseudotyped and eGFP expressing HIV-1 NL4.3 viruses encoding both Nef and Vpu (NL4.3), Vpu alone (dNef), Nef alone (dVpu), or neither accessory protein (dVpu dNef). Infected Sup-T1 cells were analyzed by flow cytometry after staining for cell surface CD28 (Fig. 1). Upon gating on live (Zombie Red^TM−^), infected (GFP^+^) single cells (Additional file 1), significantly greater cell surface levels of CD28 were present on cells infected with viruses lacking Nef (dNef), Vpu (dVpu) or both Nef and Vpu (dVpu dNef), compared to cells infected with virus encoding both Nef and Vpu (NL4.3; Fig. 1a–c). Accordingly, Western blot analysis confirmed the presence or absence of Nef and Vpu in infected Sup-T1 cells (Fig. 1d). Moreover, we examined the ability of Nef and Vpu expressed from replication competent NL4.3 provirus to downregulate cell surface CD28 in infected CD4^+^ T cells purified from peripheral blood mononuclear cells (PBMCs) (Fig. 1e, f; Additional file 2). In line with our observations in Sup-T1 cells, in the absence of Nef (dNef), Vpu (dVpu) or both Nef and Vpu (dVpu dNef) greater levels of cell surface CD28 were present on infected (p24^+^) cells compared to cells infected with NL4.3, indicating that Nef and Vpu independently downregulate cell surface CD28 in both primary CD4^+^ T cells and Sup-T1 cells.

**Fig. 2:**
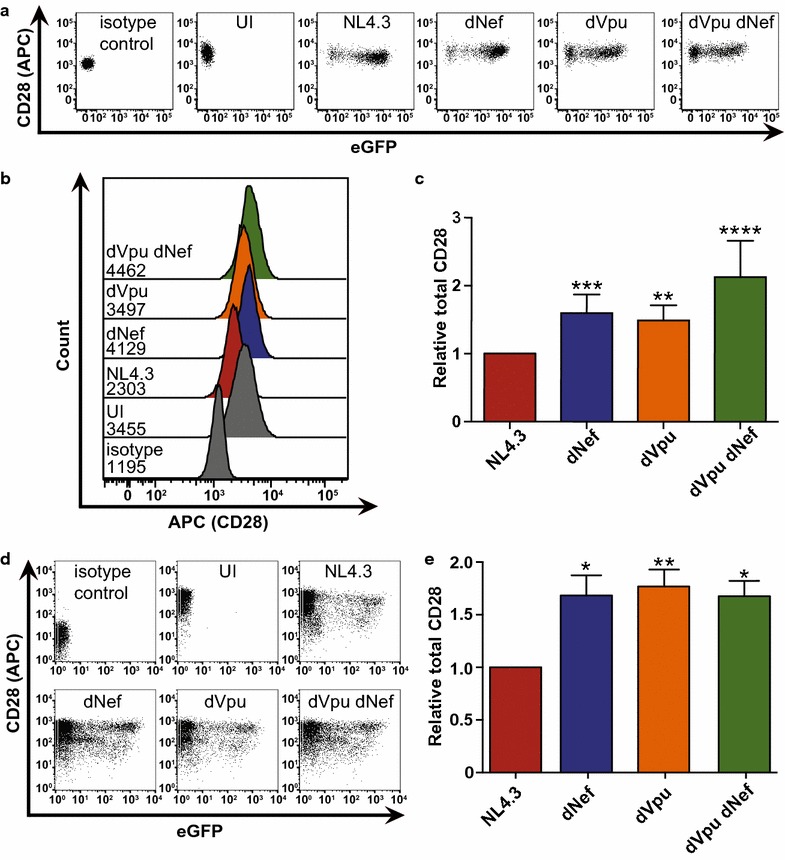
HIV-1 Nef and Vpu downregulate total CD28 protein levels. CD4^+^ Sup-T1 cells or primary CD4^+^ T cells were infected with VSV-G pseudotyped NL4.3. Viruses encoded Nef and Vpu (NL4.3, red), lacked Nef (dNef, blue), lacked Vpu (dVpu, orange) or lacked both Nef and Vpu (dNef dVpu, green). Forty-eight hours post-infection, cells were permeabilized, stained for CD28 and analyzed by flow cytometry. **a** Representative dot plots illustrating total CD28 (APC) and infection (GFP) levels of live (Zombie Red^TM−^) Sup-T1 cells. **b** Representative histograms illustrating total levels of CD28 (APC) after gating on live (Zombie Red^TM−^) infected (GFP^+^) cells. Geometric mean fluorescence intensities (MFI) are indicated. **c** Summary of mean (± SE) relative total CD28 based on MFI (n = 12). **d** Representative dot plots illustrating purified CD4^+^ T cell infection (GFP) and total CD28 (APC) levels. **e** Summary of mean (± SE) total CD28 levels on infected (GFP^+^) CD4^+^ T cells (n = 5). Data were obtained from infection of cells obtained from two healthy donors. (UI: uninfected; MFI: geometric mean fluorescent intensity; SE: standard error; *p ≤ 0.05; **p ≤ 0.01; ***p ≤ 0.001; ****p ≤ 0.0001)

We next sought to examine the effects of Nef and Vpu on total CD28 protein levels. Sup-T1 cells or CD4^+^ T cells purified from healthy donor PBMCs were infected with VSV-G pseudotyped HIV-1 NL4.3 viruses, as in Fig. 1. Infected cells were permeabilized and stained for total CD28, and subsequently analyzed by flow cytometry (Fig. 2). As with cell surface CD28, in Sup-T1 cells the total CD28 levels were significantly higher in cells infected with viruses lacking Nef (dNef) or Vpu (dVpu), relative to cells infected with viruses encoding both viral proteins (NL4.3; Fig. 2a–c). The highest total CD28 levels were observed in cells infected with virus lacking functional *nef* and *vpu* genes (dVpu dNef; Fig. 2a–c). Furthermore, the ability of Nef and Vpu to downregulate total CD28 protein was consistent in primary cells. Indeed, infection of CD4^+^ T cells purified from PBMCs with VSV-G pseudotyped NL4.3 demonstrated that cells infected with virus lacking either (dNef, dVpu) or both (dVpu dNef) viral proteins had significantly higher mean levels of CD28, compared to cells infected with virus encoding both Nef and Vpu (NL4.3) (Fig. 2d, e). This data suggests that similar to the cell surface receptor CD4 [32, 38], both Nef and Vpu downregulate total cellular CD28 protein levels.

**Fig. 3:**
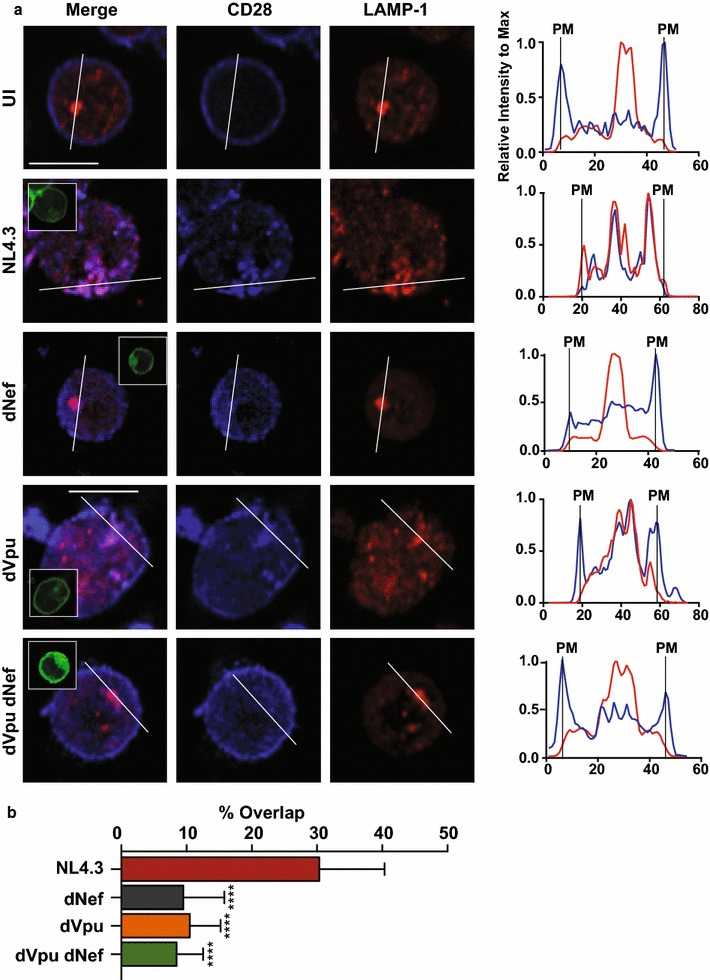
The subcellular localization of CD28 is altered in the presence of Nef or Vpu. CD4^+^ Sup-T1 cells were infected with VSV-G pseudotyped NL4.3 viruses encoding or lacking Nef and/or Vpu. Infected cells were stained for CD28 and the lysosomal marker LAMP-1 and visualized by widefield microscopy. **a** Shown are representative infected (GFP^+^) cells (left) and a graphical representation of CD28 (blue) and LAMP-1 (red) fluorescence intensity relative to the maximum along the illustrated line (right). The scale bar indicates 10 μm and the vertical lines labelled PM indicate where the illustrated line meets the plasma membrane (PM). Insets in the merged panel illustrate the GFP channel. **b** The percentage overlap between CD28 and LAMP-1 is calculated based on the mean (± SD) Manders’ overlap coefficients from at least 30 cells and 3 independent experiments. (UI: uninfected; LAMP-1: Lysosomal-associated membrane protein 1; SD: standard deviation; ****p ≤ 0.0001)

To confirm that the HIV-1 accessory proteins Nef and Vpu altered endogenous CD28, we infected Sup-T1 cells with VSV-G pseudotyped NL4.3 viruses encoding or lacking the viral proteins and examined CD28 localization using widefield microscopy (Fig. 3). Specifically, due to our observed decreases in total CD28 protein levels (Fig. 2), we stained cells for endogenous CD28 and lysosomal-associated membrane protein 1 (LAMP-1), a marker of the degradative lysosomal compartment. Interestingly, we observed that in the presence of Nef and Vpu (NL4.3) CD28 localized away from the cell surface, inconsistent with the plasma membrane localization observed in uninfected cells (Fig. 3a). Moreover, upon elimination of the viral proteins (dVpu dNef) we observed a rescue in CD28′s localization to the cell surface (Fig. 3a). Upon quantification of CD28: LAMP-1 co-localization, we found that in the presence of both viral proteins (NL4.3) the co-localization of CD28 with LAMP-1 was significantly greater than in the absence of either (dNef, dVpu) or both (dVpu dNef) viral proteins (Fig. 3b). This microscopy analysis confirms that Nef and Vpu downregulate endogenous CD28 and suggests this downregulation results in transport of CD28 to the degradative lysosome.

### Nef and Vpu-mediated effects on CD28 are dependent on the cellular degradation machinery

**Fig. 4:**
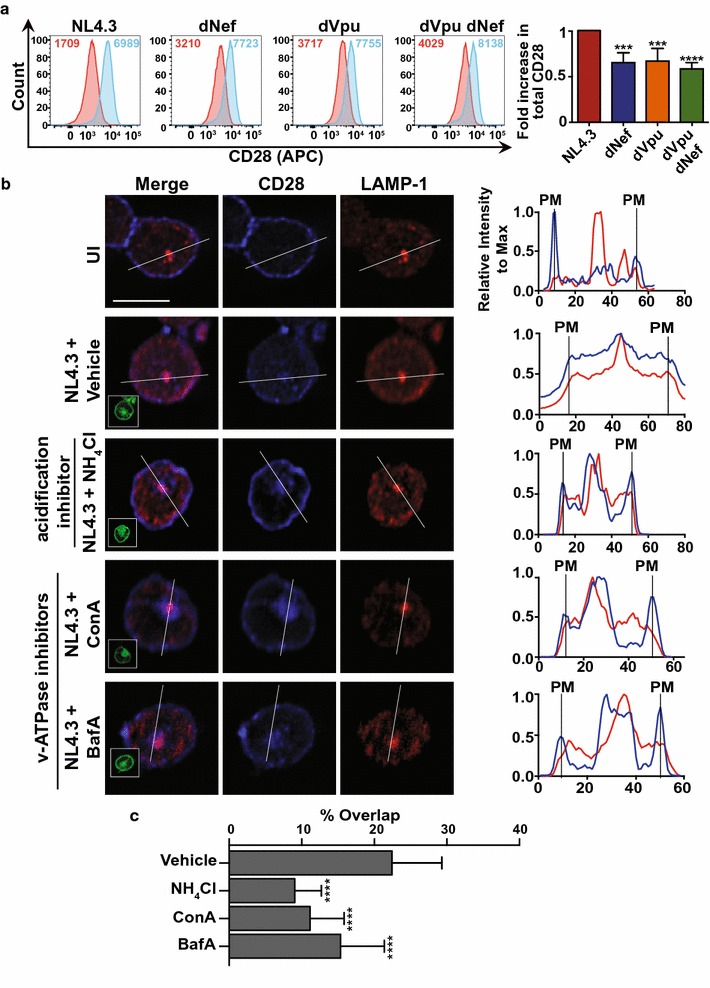
Inhibition of the degradation machinery increases CD28 protein levels in infected cells. CD4^+^ Sup-T1 cells were infected with VSV-G pseudotyped NL4.3 encoding or lacking Nef and/or Vpu. Infected cells were analyzed 48 h post-infection after treatment for 24 h with 40 mM ammonium chloride or treatment for 5 h with 100 nM Bafilomycin A1 or 100 nM Concanamycin A. Cells were stained for CD28 and the lysosomal marker LAMP-1 and analyzed by widefield microscopy or stained for CD28 and analyzed by flow cytometry. **a** Representative histograms illustrating total CD28 levels of infected cells treated with complete RPMI with (blue) or without (red) 40 mM ammonium chloride. Geometric mean fluorescence intensities (MFIs) are indicated. MFIs of infected cells were determined after gating on live (Zombie Red^TM−^), infected (GFP^+^) cells and the relative fold increase (± SE) in total CD28 upon ammonium chloride (n ≥ 5) treatment is illustrated (right). **b** Representative infected (GFP^+^) Sup-T1 cells subjected to treatment with vehicle (DMSO), ammonium chloride (NH_4_Cl), Concanamycin A (ConA) or Bafilomycin A1 (BafA) and visualized by widefield microscopy (left). The scale bar indicates 10 μm and insets in merged panel illustrate GFP channel. A graphical representation of CD28 and LAMP-1 fluorescence intensity relative to max along the illustrated line is shown (right). The vertical lines labelled PM indicate where the illustrated line meets the plasma membrane (PM). **c** The percentage of overlap between CD28 and LAMP-1 is based on the mean (± SD) Manders’ overlap coefficients measured on at least 30 cells from 3 independent experiments (UI: uninfected; SD: standard deviation; LAMP-1: Lysosomal-associated membrane protein; MFI: geometric mean fluorescent intensity; ***p ≤ 0.001; ****p ≤ 0.0001)

Cellular protein levels can be reduced via degradation by acidic hydrolases within acidified endosomal compartments [41]. Since we observed the co-localization of CD28 and LAMP-1 in infected cells (Fig. 3), we sought to determine if the Nef- and Vpu-mediated reduction in total CD28 could be blocked by inhibition of endosomal acidification. To test this, we treated infected Sup-T1 cells with the base ammonium chloride to increase intra-vesicular pH [42]. We specifically measured total CD28 within Sup-T1 cells infected with pseudotyped NL4.3 and treated with ammonium chloride prior to staining. Interestingly, upon ammonium chloride treatment, total CD28 detected by flow cytometry increased relative to untreated cells (Fig. 4a). Moreover, the fold increase, or recovery, of total CD28 protein levels upon ammonium chloride treatment was significantly lower when cells were infected with viruses lacking either (dNef, dVpu) or both Nef and Vpu (dVpu dNef), compared to Nef- and Vpu-encoding virus (NL4.3; Fig. 4a). In addition, we examined the effects of ammonium chloride on the CD4 receptor (Additional file 3). Similar to CD28, the recovery of CD4 levels upon ammonium chloride treatment was significantly less in cells infected with virus lacking one or both of the accessory proteins (dNef, dVpu, dVpu dNef; Additional file 3). Therefore, in the presence of the viral accessory proteins Nef and Vpu, a decrease in total CD28 occurs and this effect is mitigated by inhibition of endosomal acidification.

To further confirm these findings, we used widefield microscopy to visualize CD28 within Sup-T1 cells infected with VSV-G pseudotyped NL4.3. Specifically, we examined the subcellular localization of CD28 within infected cells treated with the general endosome acidification inhibitor ammonium chloride or treated with Bafilomycin A1 or Concanamycin A, specific inhibitors of the endosome acidifying proton pump, the vacuolar ATPase [43, 44]. We observed that upon treatment with ammonium chloride, Bafilomycin A1, or Concanamycin A, the distribution of CD28 within VSV-G pseudotyped NL4.3 infected cells was altered relative to vehicle (Dimethyl sulfoxide (DMSO)) treated cells (Fig. 4b). Indeed, inhibitor treatment resulted in the increased localization of CD28 to the cell surface when compared to cells treated with vehicle. Moreover, quantification of the co-localization of CD28 and LAMP-1 in Bafilomycin A1 or Concanamycin A treated cells indicated that upon inhibitor treatment CD28 co-localizes significantly less with the lysosomal marker LAMP-1 (Fig. 4c). These results suggest that the ability of Nef and Vpu to downregulate CD28 is dependent on endolysosomal acidification.

### Motifs ascribed to specific Nef:host cell protein interactions are critical for Nef-mediated CD28 downregulation

**Fig. 5:**
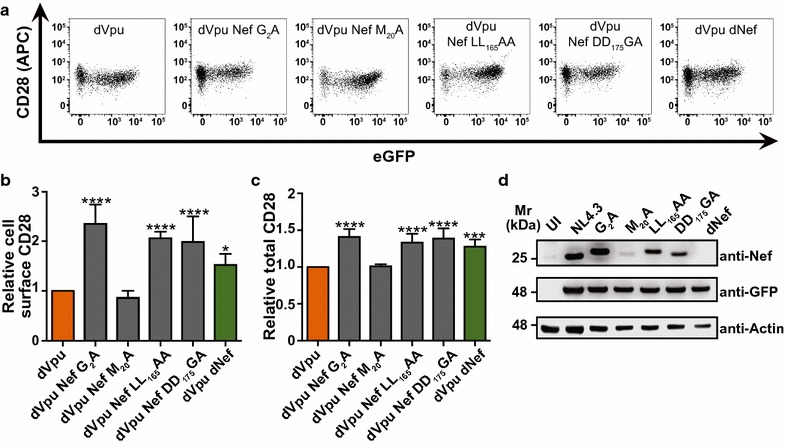
Nef:host cell protein interaction motifs are critical for Nef-mediated CD28 downregulation. Infected CD4^+^ Sup-T1 cells were stained for CD28 and analyzed by flow cytometry. Cells infected with VSV-G pseudotyped NL4.3 lacking Vpu (dVpu, orange) or Vpu and Nef (dVpu dNef, green) were used as controls. **a** Representative dot plots illustrating CD28 (APC) and infection (GFP) in live (Zombie Red^TM−^) cells. **b** Mean (± SE) relative cell surface CD28 MFIs of cells infected with NL4.3 lacking Vpu and encoding the indicated mutations in *nef* (n ≥ 4). **c** Relative mean (± SE) total CD28 within live cells infected with NL4.3 encoding various Nef mutations (n ≥ 5). **d** Western blot illustrating expression of the mutated Nef proteins. (Mr: molecular weight; UI: uninfected; SE: standard error; MFI: geometric mean fluorescent intensity; *p ≤ 0.05; ***p ≤ 0.001; ****p ≤ 0.0001)

To further elucidate the mechanisms used by Nef to downregulate CD28, we sought to determine if this function is dependent on motifs within Nef that have been previously implicated in interacting with membrane trafficking regulators that act to downregulate other cell surface receptors. Accordingly, Sup-T1 cells were infected with isogenic Gag-Pol truncated VSV-G pseudotyped NL4.3 viruses that only differed at the following Nef motifs: the myristoylation site (G_2_; [45]), the methionine residue critical for Nef:AP-1:MHC-I complex formation (M_20_; [22]), the dileucine motif implicated in adaptor protein-complex formation (LL_165_; [46]) and the diacidic motif implicated in vacuolar ATPase binding (DD_175_; [47]). Specifically, Sup-T1 cells were infected with pseudotyped NL4.3 encoding a non-functional Vpu protein and the various Nef mutants. Subsequently, live and infected single cells were analyzed (Additional file 1). In addition, cells were infected with pseudotyped NL4.3 encoding a functional Vpu protein and the various Nef mutants (Additional files 4, 5). As expected, cells infected with virus encoding Nef G_2_A [45], which disrupts most known Nef functions, exhibited significantly higher cell surface levels of CD28 compared to cells infected with virus encoding wild-type Nef (Fig. 5a, b; Additional file 5). Interestingly, cells infected with viruses encoding the LL_165_AA and DD_175_GA Nef mutations also showed significantly higher cell surface levels of CD28, suggesting that the LL_165_ and DD_175_ motifs are critical for cell surface CD28 downregulation (Fig. 5a, b; Additional file 5). Conversely, mutation of M_20_ (M_20_A), a residue essential for the downregulation of MHC-I via the interaction with AP-1 [22, 48], did not affect CD28 cell surface downregulation (Fig. 5a, b; Additional file 5). Importantly, the dileucine (LL_165_AA) and diacidic (DD_175_GA) motif mutations did not inhibit other Nef functions, as these mutated Nef proteins retained the ability to downregulate cell surface MHC-I (Additional file 5), demonstrating that the dileucine and diacidic motifs function in downregulating CD28.

In parallel, Sup-T1 cells were infected with the equivalent viruses from Fig. 5b and total CD28 levels were measured by flow cytometry. The observed decrease in total CD28 in the presence of Nef (Fig. 2) was abolished by mutation of the myristoylation site (G_2_A) and the diacidic motif (DD_175_GA), as cells infected with viruses encoding these mutated Nef proteins exhibited significantly higher total levels of CD28 compared to those infected with virus encoding wild-type Nef (Fig. 5c; Additional file 5). However, unlike cell surface CD28, the total CD28 levels did not differ significantly between cells infected with virus encoding wild-type Nef versus Nef LL_165_AA in cells infected with viruses also encoding Vpu (Additional file 5). Contrastingly, in cells infected with virus lacking Vpu and encoding the Nef LL_165_AA mutation, we observed significantly greater levels of total CD28 (Fig. 5c). Finally, expression of all mutated Nef proteins was confirmed by Western blot (Fig. 5d). Taken together, these findings implicate the Nef diacidic motif (DD_175_) in the downregulation of total and cell surface CD28.

### Motifs in Vpu implicated in interactions with specific host cellular proteins are critical for CD28 downregulation

**Fig. 6:**
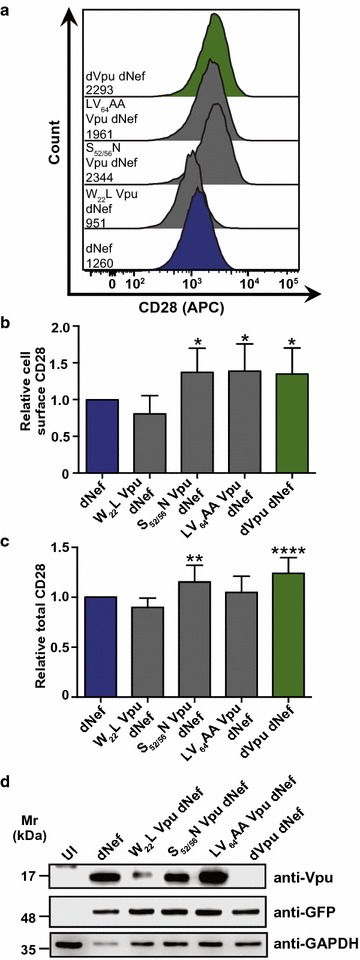
Motifs in Vpu are critical for downregulation of CD28. Infected CD4^+^ Sup-T1 cells were stained for CD28 and analyzed by flow cytometry. Mean geometric fluorescence intensities (MFI) of cells were determined after gating on live and infected (Zombie Red^TM−^ and GFP^+^) cells. Cells infected with VSV-G pseudotyped NL4.3 lacking Nef (dNef, blue) and both Nef and Vpu (dNef dVpu, green) were used as controls. **a** Representative histograms illustrating cell surface CD28 on live (Zombie Red^TM−^) infected (GFP^+^) cells. MFIs are indicated. **b** Mean (± SE) relative cell surface CD28 on cells infected with viruses encoding mutations in *vpu* (n ≥ 9). **c** Relative mean (± SE) total CD28 within cells infected with viruses encoding the indicated *vpu* mutations (n ≥ 7). **d** Western blot illustrating expression of mutated Vpu proteins. (Mr: molecular weight; UI: uninfected; SE: standard error; GAPDH: glyceraldehyde 3-phosphate dehydrogenase; MFI: geometric mean fluorescent intensity; GFP: green fluorescent protein; *p ≤ 0.05; **p ≤ 0.01; ****p ≤ 0.0001)

Next, we sought to gain insight into the mechanism used by Vpu to downregulate CD28 by examining the ability of mutant Vpu proteins to modulate cell surface and total CD28 levels. Mutated motifs within the NL4.3 *vpu* gene which have been previously implicated in the ability of Vpu to downregulate CD4 [49–51] or the restriction factor BST-2 [52–54] were inserted into a NL4.3 viral backbone encoding eGFP, but lacking a functional *nef* gene (pNL4.3 dG/P eGFP dNef). Sup-T1 cells were infected with these VSV-G pseudotyped isogenic NL4.3 viruses, which only differ in the specific *vpu* mutations. Relative to cells infected with virus encoding wild-type Vpu (dNef), significantly greater levels of cell surface CD28 were present on cells infected with viruses encoding Vpu mutations at the adaptor protein interaction interface (LV_64_AA Vpu dNef) and serine residues (S_52/56_N Vpu dNef) which are phosphorylated by casein kinase II to facilitate recruitment of E3 ubiquitin ligase complex components (Fig. 6a, b) [38]. In contrast, relative to cells infected with virus encoding wild-type Vpu (dNef), cell surface CD28 levels were not significantly different on cells infected with virus encoding a mutation within a Vpu transmembrane domain residue implicated in the downregulation of CD4 and BST-2 (W_22_A Vpu dNef [50, 53]; Fig. 6a, b). The functionality of the mutant Vpu proteins was tested by examining the effects of the mutations on Vpu-mediated CD4 downregulation in Sup-T1 cells (Additional file 6). Indeed, upon infection with viruses encoding the W_22_L, S_52/56_N or LV_64_AA mutations, significantly greater cell surface levels of CD4 were present relative to cells infected with virus encoding wild-type Vpu (dNef) (Additional file 6), suggesting that the W_22_ motif is critical for CD4, but not cell surface CD28 downregulation.

In parallel, viruses encoding mutated Vpu proteins were also tested for their effects on the downregulation of total CD28. Total CD28 levels were not significantly different when comparing cells infected with virus encoding wild-type Vpu or the W_22_L or LV_64_AA mutations (Fig. 6c). In contrast, there was significantly greater total CD28 in the absence of Vpu (dVpu dNef) and in the presence of Vpu encoding the S_52/56_N mutation (S_52/56_N dNef) relative to virus encoding wild-type Vpu (dNef) (Fig. 6c). As an additional control, we demonstrated that cells infected with virus encoding the W_22_L, S_52/56_N or LV_64_AA mutations exhibited significantly greater total levels of CD4 relative to cells infected with virus encoding wild-type Vpu (Additional file 6). Expression of all mutated Vpu proteins was confirmed by Western blot (Fig. 6d). As with our findings for cell surface CD28 downregulation, our results suggest that the molecular determinants of CD28 downregulation by Vpu are distinct from those utilized to downregulate CD4.

### Patient-derived Vpu proteins mediate CD28 downregulation

**Fig. 7:**
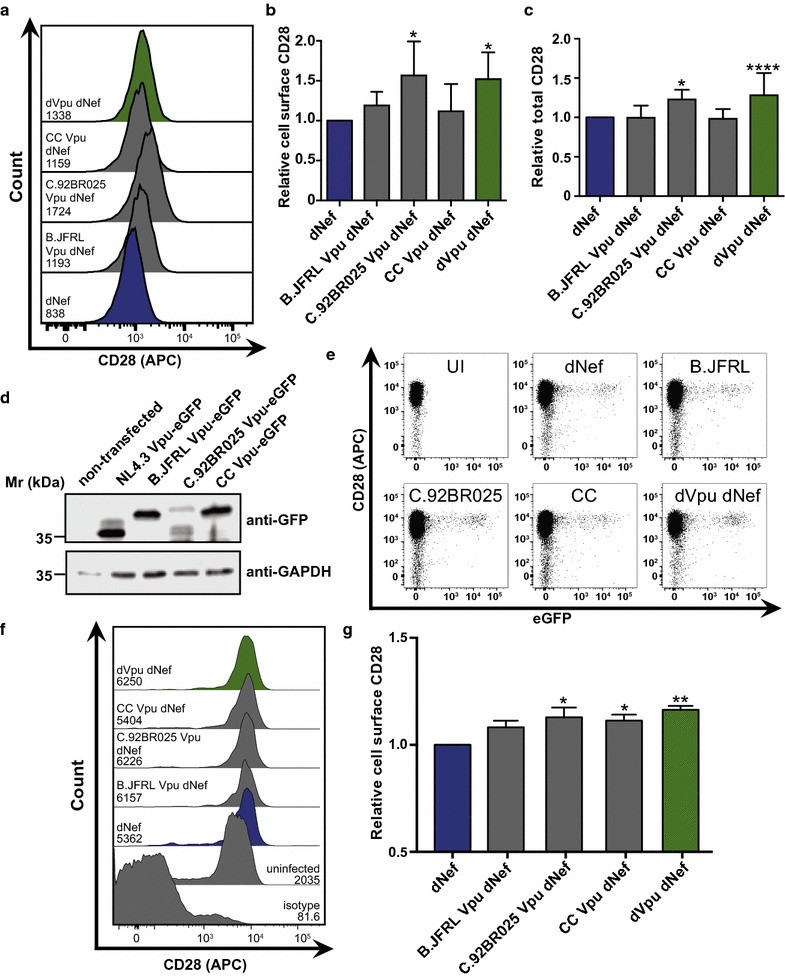
Non-NL4.3 Vpu proteins downregulate cell surface CD28. CD4^+^ Sup-T1 and peripheral blood mononuclear cells (PBMCs) were infected with VSV-G pseudotyped NL4.3 viruses lacking Nef and encoding wild-type Vpu (dNef, blue), no Vpu (dNef dVpu, green) or three non-NL4.3 Vpu proteins (grey). Cells were analyzed by flow cytometry and mean geometric fluorescence intensities (MFI) of infected (GFP^+^) cells were determined. **a** Representative histograms illustrating cell surface CD28 on live infected (GFP^+^) Sup-T1 cells. MFIs are indicated. **b** Mean (± SE) relative cell surface CD28 on Sup-T1 cells infected with NL4.3 lacking a functional Nef protein (dNef), but encoding various Vpu proteins (n = 5). **c** Mean (± SE) relative total cellular CD28 of Sup-T1 cells infected with NL4.3 encoding various Vpu proteins (n ≥ 8). **d** Western blot illustrating expression of eGFP-tagged versions of the analyzed Vpu proteins from transfected HEK293T cells. **e** Representative dot plots illustrating infection level (GFP) and cell surface CD28 (APC) on primary CD4^+^ cells. **f** Representative histograms illustrating cell surface CD28 on either uninfected (isotype control, uninfected) or CD4^+^ infected (GFP^+^) cells. MFIs are indicated. **g** Mean (± SE) relative cell surface CD28 on CD4^+^ cells infected with viruses encoding various *vpu* genes (n = 8). Data were obtained from infection of PBMCs from two healthy donors. (Mr: molecular weight; GAPDH: glyceraldehyde 3-phosphate dehydrogenase; GFP: green fluorescent protein; SE: standard error; UI: uninfected; *p ≤ 0.05; **p ≤ 0.01; ****p ≤ 0.0001)

Our findings suggest that HIV-1 NL4.3 can downregulate CD28 by both Nef and Vpu. However, as HIV-1 is genetically diverse, functions observed in the laboratory strain NL4.3 may not necessarily play a prominent role in the epidemic at large. We therefore wanted to determine if Vpu-mediated CD28 downregulation is a conserved function. Thus, we tested the ability of multiple group M Vpu proteins to downregulate cell surface and total CD28. To test this, the NL4.3 *vpu* gene from a Gag-Pol truncated NL4.3 virus lacking Nef (NL4.3 dG/P eGFP dNef) was replaced with *vpu* genes from an HIV-1 subtype B reference strain (B.US.86.JRFL), a subtype C reference strain (C.BR.92.92BR025; [55]), or a gene encoding a subtype C consensus protein (CC). Sup-T1 cells were infected with these VSV-G pseudotyped isogenic viruses which differed only in the encoded Vpu proteins, and cell surface and total CD28 levels were measured by flow cytometry (Additional file 1). Cells infected with virus encoding a subtype C consensus (CC) Vpu protein or a subtype B reference strain (B.US.86.JRFL) Vpu protein did not significantly differ in cell surface levels of CD28 relative to cells infected with virus encoding NL4.3 Vpu (Fig. 7a, b), suggesting that these additional Vpu proteins were capable of downregulating cell surface CD28. In contrast, cells infected with virus encoding a subtype C reference strain (C.BR.92.92BR025) *vpu* gene had significantly greater cell surface levels of CD28, as with virus lacking a functional *vpu* gene (dVpu dNef) (Fig. 7a, b). Moreover, staining for total cellular CD28 revealed that cells infected with virus encoding the B.US.86.JRFL and consensuses C *vpu* genes did not differ in total cellular CD28 levels relative to cells infected with virus encoding NL4.3 *vpu*, while the C.BR.92.92BR025 Vpu protein did not downregulate total CD28 (Fig. 7c). Expression of these proteins in a heterologous system revealed equivalent protein levels for all Vpu proteins except for Vpu from C.BR.92.92BR025, which is weakly expressed (Fig. 7d). To confirm that these findings were not unique to the Sup-T1 cell line, PBMCs were infected with the same pseudotyped NL4.3 viruses (Fig. 7a) and cell surface CD28 levels on infected (GFP^+^) CD4^+^ cells was measured (Additional file 7). A similar trend to that observed in Sup-T1 cells was noted, as the mean levels of CD28 expression were significantly greater on cells infected with virus lacking the *vpu* gene, than in cells infected with virus encoding the NL4.3 and B.US.86.JRFL *vpu* genes (Fig. 7e–g). However, unlike Sup-T1 cells, cell surface levels of CD28 were significantly higher on cells infected with virus encoding the consensus C (CC) Vpu protein, than the NL4.3 Vpu protein. Overall, this suggests that Vpu-mediated cell surface and total CD28 downregulation is not limited to the laboratory adapted HIV-1 strain NL4.3.

### Nef and Vpu expression alters the cellular response to external CD28 stimulation

**Fig. 8:**
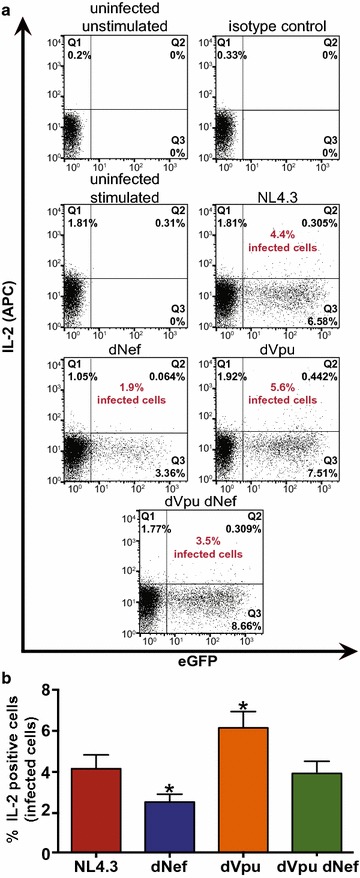
Response of infected cells to CD28-stimulation is altered in the presence of Nef or Vpu. Purified CD4^+^ T cells were infected with VSV-G pseudotyped NL4.3 encoding Nef and Vpu (NL4.3, red), lacking Nef (dNef, blue) or Vpu (dVpu, orange), or lacking both Nef and Vpu (dVpu dNef, green). Twenty-four hours post-infection cells were activated with anti-CD3/anti-CD28 for 24 h. Cells were then incubated with Brefeldin A for 12 h and stained for intracellular IL-2 prior to analysis by flow cytometry. **a** Representative dot plots illustrating intracellular IL-2 (APC) and infection (GFP) levels. Quadrants were selected based on the IL-2 isotype antibody control and uninfected controls. The percentage of infected cells that are IL-2 positive is indicated (red). The percentage of cells making up the uninfected and IL-2 negative population (Q4) are as follows: uninfected unstimulated: 99.8%, isotype control: 99.7%, uninfected stimulated: 93.8%, NL4.3: 91.3, dNef: 95.5%, dVpu: 90.1, dVpu dNef: 89.3%. **b** Mean (± SE) percentage of infected cells that are IL-2 positive (n ≥ 6). The means were obtained by analysis of infected cells from two healthy donors. (SE: standard error; *p ≤ 0.05)

Given that HIV-1 decreases cell surface levels of CD28 (Fig. 1), a key immune cell stimulatory receptor, we postulated that decreased cell surface levels of CD28 on infected cells leads to a decreased ability of cells to become activated by CD28/CD3 stimulation. Thus, we tested what effects Nef and Vpu may have on activation in CD4^+^ T cells stimulated by CD28/CD3. We therefore infected primary CD4^+^ T cells with VSV-G pseudotyped NL4.3 encoding both Nef and Vpu (NL4.3), Nef alone (dVpu), Vpu alone (dNef) or lacking both Nef and Vpu (dVpu dNef). Infected cells were stimulated with soluble anti-CD28 and plate bound anti-CD3, and activation was determined by measuring intracytoplasmic production of IL-2, an event that occurs downstream from CD28 activation [56] (Fig. 8). Upon infection with virus lacking Nef (dNef), the percentage of infected cells that produced IL-2 after stimulation was significantly lower than in cells infected with virus encoding Nef and Vpu (NL4.3) (Fig. 8a, b), suggesting that the presence of Nef leads to increased activation, consistent with Nef’s previously reported role in T cell activation upon anti-CD3 and anti-CD28 treatment of Jurkat cells [57]. In contrast, in primary cells infected with a virus lacking Vpu (dVpu), the proportion of IL-2 positive infected cells was significantly increased, suggesting that Vpu decreases anti-CD3/CD28 dependent cell activation. This increased activation in the presence of Nef and decreased activation in the presence of Vpu was also evident in experiments conducted with virus lacking both Nef and Vpu (dVpu dNef), as the proportion of activated cells did not differ from cells infected with wild-type virus (NL4.3; Fig. 8a, b). Overall, this suggests that Nef and Vpu, which influence cell surface and total CD28 levels, differentially regulate responsiveness to CD28 stimulation.

## Discussion

In the current report, we describe the distinct ability of both HIV-1 Nef and Vpu to decrease cell surface and total levels of CD28 in CD4^+^ T cells. Specifically, we have shown cell surface CD28 downregulation with VSV-G pseudotyped NL4.3 in Sup-T1 cells and primary cells, as well as downregulation in primary CD4^+^ T cells infected with replication competent NL4.3 (Figs. 1, 7). These results are consistent with observations made in cell lines transiently transfected with Nef and/or Vpu expression vectors. Downregulation of endogenous CD28 was previously illustrated in Jurkat cells transiently expressing Nef [40]. Moreover, Haller et al. completed a large screen for receptors downregulated by Nef and Vpu through transient expression of the viral proteins in A3.01 T lymphocytes, and observed downregulation of CD28 by both HIV-1 Nef and Vpu [20]. Interestingly, the latter study demonstrated varying degrees of downregulation by HIV-1 Nef and Vpu, as well as SIV_mac239_ Nef, with respect to the multiple tested receptors [20]. Accordingly, we observed that the effects of Nef and Vpu on CD28 are subtler than the effects of Nef and Vpu on other receptors, namely MHC-I and CD4 (Additional file 5, 6), but nevertheless Nef and Vpu traffic this receptor to a degradative lysosomal compartment and decrease total CD28 protein levels (Figs. 2, 3, 4). Moreover, it has been shown that there are varying degrees of CD28 downregulation by various Nef proteins, with HIV-2 and certain SIV derived Nef proteins exhibiting more efficient downregulation than HIV-1 Nef proteins [20, 58]. However, this does not limit the potential physiological relevance of CD28 downregulation by HIV-1 and our current report demonstrates the mechanistic details of Nef and Vpu-mediated CD28 downregulation.

Our results demonstrate that slight increases in cell surface CD28 levels can be observed in cells infected with viruses lacking Nef and Vpu (dVpu dNef) in comparison to uninfected cells (Fig. 1b, e). This suggests that HIV-1 proteins other than Nef and Vpu may also be contributing to changes in cell surface CD28 levels upon infection. The latter effects may be due to events independent of membrane trafficking and could be the consequence of changes at the level of CD28 transcription. Notably, CD28 expression levels may be affected at the transcriptional level via the HIV-1 tat protein, as it interacts with the Sp1 transcription factor which positively affects CD28 transcription [59, 60].

Given that Nef and Vpu collaborate to mediate the downregulation and degradation of cellular receptors, specifically CD4 [28, 61–63], we hypothesized that the observed decreases in total CD28 levels in the presence of Nef and Vpu were due to CD28 degradation. This is supported by our observation that CD28 co-localizes with the lysosome marker LAMP-1 in the presence of the viral proteins (Fig. 3). Moreover, we found that we could limit Nef- and Vpu-mediated decreases in total CD28 and CD4 protein levels upon treatment with ammonium chloride (Fig. 4; Additional file 3). The effects of ammonium chloride on CD4 downregulation are of interest given the previously reported Vpu-dependent proteasomal degradation of CD4 [64]. In our experiments, ammonium chloride may be playing a broader role and impacting cellular physiology by affecting general cell acidification. Importantly, we have used the specific endosomal acidification inhibitors Bafilomycin A1 and Concanamycin A to provide a more specific mechanistic explanation of Nef- and Vpu-dependent effects on CD28 (Fig. 4), thereby bypassing the more general effects of using ammonium chloride. The specific inhibition of the vacuolar ATPase with these molecules limits the co-localization of CD28 with LAMP-1 in Nef and Vpu-expressing cells (Fig. 4).

We also report how CD28 depletion involves the distinct ability of Nef and Vpu to interact with host cellular proteins. Indeed, cell surface CD28 downregulation was inhibited by mutation of Nef LL_165_ and DD_175_, while maintaining the ability to downregulate MHC-I (Fig. 5; Additional file 5). The findings that Nef LL_165_ and DD_175_ are critical for cell surface CD28 downregulation is in accordance with previously published reports [40, 65], and suggests that AP-2 [46] and the vacuolar ATPase [47] are critical for cell surface downregulation by Nef. Interestingly, mutation of the G_2_, LL_165_ and DD_175_ motifs abolished Nef-dependent decreases in total levels of cellular CD28 in the absence of Vpu (Fig. 5), while mutation of the LL_165_ motif did not significantly alter total CD28 levels in the presence of Vpu (Additional file 5). This may be due to Vpu’s ability to compensate for specific Nef mutations. Our observed increases in total cellular CD28 when the ability of Nef to interact with the vacuolar ATPase is inhibited further supports the idea that Nef mediates degradation of CD28 (DD_175_GA; Fig. 5c). The Nef DD_175_ motif is also critical for Nef-mediated lysosomal degradation of the co-inhibitory receptor Cytotoxic T-Lymphocyte-Associated protein 4 (CTLA-4) [66], which interestingly, competes with CD28 for binding the same receptors, B7.1/B7.2 [67]. Overall, this suggests that as with cell surface downregulation, the downregulation of total CD28 levels is dependent on specific Nef:host cell protein interaction motifs.

We have also provided mechanistic details on how Vpu modulates CD28 expression and localization within HIV-1 infected cells. Intriguingly, the mutations exhibited different effects on CD28 and CD4 downregulation (Fig. 6; Additional file 6), indicating that CD28 downregulation by Vpu may have evolved independently. Specifically, mutation of the Vpu W_22_, S_52/56_ and LV_64_ motifs inhibited the ability of Vpu to downregulate cell surface CD4 (Additional file 6), in agreement with previous reports [49–51]. In contrast, mutation of the highly conserved W_22_ did not inhibit cell surface downregulation of CD28 (Fig. 6a, b), suggesting that this residue is dispensable for CD28 downregulation despite being implicated in the downregulation of CD4 and BST-2 [50, 53, 68, 69].

Similar to CD4 downregulation, mutation of Vpu S_52_/S_56_ and LV_64_ inhibited cell surface downregulation of CD28 (Fig. 6a, b). Interestingly, these Vpu motifs play distinct roles with AP-1 and AP-2, in addition to contributing to mechanisms that promote the degradation of cellular proteins. Indeed, the non-phosphorylatable mutants of Vpu (Vpu S_52/56_N) do not recruit AP-1 and AP-2 and subsequently fail to downregulate BST-2 [37], whereas Vpu LV_64_ promotes the degradation of BST-2 via autophagy [70]. Thus, the ability of Vpu to downregulate CD28 from the cell surface may require AP-1 or AP-2 binding to the LV_64_ motif in a Vpu S_52_/S_56_ dependent manner to exclude CD28 from the cell surface post-endocytosis or prior to anterograde transport.

Interestingly, only the S_52/56_N mutations in Vpu inhibited Vpu-mediated decreases in total CD28, whereas the W_22_, S_52/56_ and LV_64_ motifs were all imperative to decreasing total CD4 (Fig. 6c; Additional file 6). The adaptor protein-binding motif in Vpu (LV_64_) was critical for cell surface downregulation, but dispensable for decreasing total CD28 (Fig. 6b, c). This motif may be unnecessary for reducing total CD28 levels as additional host cell binding factors, either known or currently unidentified, may compensate for mutation of the LV_64_ motif. Overall, the mechanism of Vpu-mediated CD28 cell surface downregulation is discrete from that which mediates total decreases in cellular CD28, and this mechanism is distinct from Vpu-mediated downregulation of total CD4.

We demonstrated that the ability of Vpu to downregulate cell surface and total CD28 is not specific to the laboratory adapted NL4.3 strain Vpu protein. Specifically, a subtype C consensus Vpu protein and a subtype B reference strain (B.US.86.JRFL) protein downregulated cell surface and total CD28 in infected Sup-T1 cells (Fig. 7). Contrastingly, the Vpu protein derived from a subtype C reference strain (C.BR.92.92BR025) did not downregulate CD28 (Fig. 7), which may be attributable in part to decreased expression (Fig. 7d). Interestingly, in a separate study we found that this subtype C reference strain encodes a non-functional Nef protein and has previously been shown to have lower fitness when compared to other well described subtype reference strains [71, 72]. Moreover, we found that in infected CD4^+^ PBMCs both the NL4.3 and B.US.86.JRFL derived Vpu proteins were capable of downregulating cell surface CD28 (Fig. 7e–g). However, unlike in Sup-T1 cells, PBMCs infected with virus encoding the consensus C Vpu protein did not exhibit downregulation (Fig. 7g). The observed difference between Sup-T1 cells and CD4^+^ PBMCs may be due to intrinsic differences between Sup-T1 and primary cells and potential differences in the expression pattern of the various Vpu proteins in different cell types. None the less, the ability of distinct Vpu proteins to downregulate cell surface and total CD28 suggests that this function is conserved to some extent, despite high HIV-1 diversity [73].

HIV-1 has evolved multiple means of downregulating CD28, which are in part genetically separable from CD4 and MHC-I downregulation, implying that CD28 downregulation is critical during infection. Moreover, we hypothesized that the function of CD28 downregulation is relevant during infection and that CD28 downregulation by Nef and Vpu may alter cell activation through CD28 receptor stimulation. Indeed, we observed that in primary CD4^+^ T cells infected with pseudotyped virus encoding Vpu alone (dNef), a significantly smaller proportion of infected cells secreted IL-2 upon CD3/CD28 stimulation, relative to cells infected with virus encoding Nef and Vpu (NL4.3; Fig. 8b). In contrast, cells infected with virus lacking Vpu (dVpu) displayed an increase in cell activation (Fig. 8), indicating that Nef alone increases CD28 stimulation responsiveness.

The observed differences in cell activation in the presence or absence of Nef and Vpu may be partially attributable to other reported functions of these viral proteins. Namely, Nef alters the subcellular localization of the T cell receptor and immunological synapse associated kinase Lck, leading to a physical separation of the T cell receptor from the immunological synapse [6, 74]. Nef also binds and activates the serine/threonine kinase p21 activated kinase 2 (PAK2), which induces T cell activation [57]. Nef thus alters the activation status of cells, perhaps inducing the correct balance between activating and inhibitory signaling, thereby enabling optimal viral replication without inducing anergy or activation induced cell death [75]. However, when compared to Nef, little is known regarding how Vpu alters T cell activation. Nonetheless, Vpu may interfere with T cell activation and IL-2 production through its ability to inhibit nuclear factor kappa-light-chain-enhancer of activated B cells (NF- κB) [76]. Ultimately, the evolution of CD28 downregulation may be one component of the multitude of alterations within infected cells that act to attain optimal levels of cell activation.

The role or function of CD28 downregulation in vivo remains elusive, however we speculate it may play a role in cell activation. CD28 is critical for T cell activation, providing co-stimulatory signalling necessary for activation upon binding to CD80:CD86 [4, 5] and in the absence of CD28, T cell receptor ligation can lead to an unresponsive, anergic state [78]. The importance of CD28 in vivo has been demonstrated through the use of anti-CD28 therapies to induce immunosuppression following transplantation, in patients with autoimmune diseases (reviewed in [79]) and during cancer immunotherapies via CD28 activation [80]. In addition, reductions in IL-2 levels, as observed in the presence of Vpu (Fig. 8), may lead to impairments in T cell survival, proliferation and function (reviewed in [81]). Interestingly, decreases in IL-2 levels are observed in HIV infected individuals [82, 83], and clinical trials have examined the benefit of administering recombinant IL-2 in combination with anti-retroviral therapy [84]. IL-2 has also been associated with reactivation of latently infected cells [85, 86]. Therefore, it is conceivable that CD28 downregulation alters responsiveness and activation of infected T cells.

Alterations in activation status of infected cells, which may in part be achieved through CD28 downregulation, could have effects in vivo during HIV-1 infection. Upon HIV-1 infection, a transcriptionally silent latent reservoir of cells is formed, which currently present the largest obstacle for achieving a HIV-1 cure [87]. A decrease in cell activation as a result of alterations in cell surface CD28 levels may allow an infected cell to enter a transcriptionally silent, latent state. Indeed, CD28 activation is capable of inducing HIV-1 transcription and replication, even in the absence of TCR activation [88]. Moreover, viral microRNA-mediated silencing of genes that contribute to cellular activation, which may include CD28 [77], have been suggested to be important for the development of latency [89]. Furthermore, CD28 activation is utilized to reactivate latent cells, as mTOR, a kinase activated downstream of CD28 signaling [90], was identified as a critical controller of HIV-1 latency [91].

## Conclusions

We illustrate that the HIV-1 Nef and Vpu accessory proteins downregulate CD28 from the cell surface and at the cellular level. This effect is observed with more than just the laboratory adapted NL4.3 strain, indicating this function is conserved. We propose that the decreases in total cellular CD28 may be potentiated by lysosomal degradation of CD28. Moreover, our analysis of Nef and Vpu mutants suggest that the Nef:vacuolar ATPase interaction and phosphorylation of Vpu S_52/56_ are both critical for downregulation of cell surface and total protein levels of this key co-stimulatory molecule. Finally, the presence or absence of Nef and Vpu modulates the ability of cells to respond to CD28-mediated stimulation.

## Methods

### DNA constructs

Viral infection plasmids used for VSV-G pseudotyped lentivirus production were engineered from the previously described pNL4.3 dG/P eGFP or pNL4.3 dG/P eGFP dNef backbones [92, 93]. To make viral constructs lacking Vpu, HIV-1_NL4-3_ *vpu/nef/UD* Deletion Mutant (p230-11; NIH-AIDS reagents catalog number 2535) was digested with EcoRI and BamHI and the fragment encoding the *vpu* gene was sub-cloned into pNL4.3 dG/P eGFP or pNL4.3 dG/P eGFP dNef. Mutations in the NL4.3 *nef* gene were produced by site directed mutagenesis within a pN1 expression vector (Clontech, Mountain View, CA) encoding NL4.3 Nef. Mutagenesis primers were created with the Agilent Technologies QuickChange Primer Design program (Agilent Technologies, Santa Clara, CA). The *nef* genes containing mutations were subsequently PCR amplified with primers encoding XmaI and NotI cut sites, and were inserted into a previously described vector encoding XmaI and NotI cut sites flanking the *nef* gene [94]. Mutations in the NL4.3 *vpu* gene were produced by site directed mutagenesis in a pN1 Vpu-FLAG expression vector. *Vpu* genes encoding mutations were subsequently PCR amplified and inserted into pRECnfl HIV-1 dVpu/URA3 (obtained from Eric Arts, University of Western Ontario), using a previously described yeast recombination system [95]. The *vpu*-encoding fragment was then sub-cloned into a NL4.3 backbone lacking a functional *nef* gene (pNL4.3 dG/P eGFP dNef) using the EcoRI and BamHI restriction sites. *Vpu* genes derived from a subtype C reference strain (C.BR.92.92BR025), subtype B reference strain (B.US.86.JRFL) or a consensus C protein synthesized using Invitrogen GeneArt Synthesis (Thermo Fisher Scientific, Mississauga, ON) were inserted into the pRECnfl HIV-1 dVpu/URA3 vector followed by sub-cloning into pNL4.3 dG/P eGFP dNef, as described above. To test the expression of the non-NL4.3 Vpu proteins the *vpu* encoding fragments were PCR amplified and sub-cloned into the peGFP-N1 expression vector (Clontech).

Viral infection plasmids utilized to make replication competent NL4.3 were engineered from the previously described pNL4.3 vector [93]. A Nef deficient plasmid (pNL4.3 dNef) was obtained from Gary Thomas (University of Pittsburgh) and Vpu (dVpu) and Nef and Vpu (dVpu dNef) deficient plasmids were produced by subcloning the *vpu* encoding portion of HIV-1_NL4-3_ *vpu/nef/UD* Deletion Mutant into pNL4.3 or pNL4.3 dNef, as described above.

### Cell culture

HEK293T (ATCC) and U87 CD4^+^ CXCR4^+^ (NIH-AIDS Research and Reference Reagent program; catalog number 4036) cells were maintained in Dulbecco’s modified Eagle’s medium (DMEM) containing 4 mM l-glutamine, 4500 mg/L glucose and sodium pyruvate (HyClone, Logan, UT) and supplemented with 1% Penicillin–Streptomycin (Hyclone) and 10% fetal bovine serum (FBS; Wisent, St-Bruno, QC). Sup-T1 cells were maintained in Roswell Park Memorial Institute media (RPMI) 1640 media with l-glutamine supplemented with 100 μg/ml penicillin–streptomycin, 1% sodium pyruvate, 1% non-essential amino acids, 2 mM l-glutamine (Hyclone) and 10% FBS. All cell lines were grown at 37 °C in the presence of 5% CO_2_ and sub-cultured in accordance with supplier’s recommendations.

Primary peripheral blood mononuclear cells were isolated from four healthy donors by density centrifugation using Histopaque (Sigma-Aldrich, Oakville, ON) and cryopreserved. Upon revival, cells were maintained in RPMI with l-glutamine supplemented with 100 μg/ml penicillin–streptomycin, 1% sodium pyruvate, 1% non-essential amino acids, 2 mM l-glutamine (Hyclone) and 10% FBS at 37 °C and 5% CO_2_ in the presence or absence of IL-2 and phytohemagglutinin (PHA) stimulation, as indicated. CD4^+^ T cells were purified from PBMCs using the MojoSort^TM^ human CD4 T cell isolation kit (BioLegend, San Diego, CA) and MACS cell separation columns (Miltenyi Biotec, Auburn, CA), according to the manufacturer’s protocol.

For ammonium chloride treatment, cells were pelleted and culture medium was replaced with complete RPMI containing or lacking 40 mM ammonium chloride (Sigma-Aldrich) in phosphate buffered saline (PBS) 24 h prior to analysis. For vacuolar ATPase inhibitor treatment, cells were pelleted and culture medium was replaced with complete RPMI containing vehicle, 100 nM Concanamycin A (Santa Cruz Biotechnology, Dallas, TX) or 100 nM Bafilomycin A1 (Sigma-Aldrich) in DMSO 5 h prior to analysis.

### Transfections and infections

For VSV-G pseudotyped lentivirus production, HEK293T cells were triply transfected with the pNL4.3 dG/P eGFP vector of interest, the VSV-G envelope-encoding pMD2.G plasmid (Addgene; catalog number 12259) and pCMV-DR8.2 (Addgene; catalog number 12263) using PolyJet (FroggaBio, North York, ON) as per the manufacturer’s protocol. Forty-eight hours post transfection, lentivirus was harvested via cell culture supernatant clarification by spinning at 1500×*g*, followed by 20 μm filtration. For production of NL4.3 provirus, viral vectors were transfected into HEK293T cells using PolyJet. Forty-eight hours post-transfection, cell supernatant was applied to U87 CD4^+^ CXCR4^+^ cells to propagate the virus. Cell supernatant was harvested 4–6 days post infection as described above. Viruses were stored in 20% FBS at − 80 °C.

For infection of Sup-T1 cells with VSV-G pseudotyped viruses, 8 × 10^5^ Sup-T1 cells were pelleted and re-suspended in the appropriate volume of pseudovirus in 20% FBS, 8 μg/mL polybrene and brought to 1 mL with 10% complete RPMI. Forty-eight hours post infection, cells were analyzed via flow cytometry, microscopy or Western blotting. For infection of peripheral blood mononuclear cells with VSV-G pseudotyped viruses, cells were cultured for 3 days in 10 ng/mL IL-2 (PeproTech, Rocky Hill, NJ) and 5 μg/mL PHA (Sigma-Aldrich). Cells were then pelleted and re-suspended in the appropriate volume of pseudovirus containing 8 μg/mL polybrene. Cells were subsequently spinoculated for 4 h at 2880×*g* at room temperature, re-suspended in fresh RPMI and incubated for 2 days prior to analysis. For infection of primary CD4^+^ T cells with replication competent NL4.3 viruses, cells were cultured for 3 days in 10 ng/mL IL-2 and 5 μg/mL PHA-L. CD4^+^ T cells were then purified as described above and re-suspended in the appropriate volume of virus in 20% FBS and 8 μg/mL polybrene. Subsequently, cells were spinoculated for 4 h at 2880×*g* at room temperature, re-suspended in fresh complete RPMI containing 5 ng/mL IL-2 and 5 μg/mL PHA-L. Two days post-infection, cells were pelleted and re-suspended in fresh media containing PHA and IL-2 and 4 days post-infection cells were fixed and stained. For infection of purified CD4^+^ T cells with VSV-G pseudotyped virus, cells were cultured for 3 days in 10 ng/mL IL-2 and 5 μg/mL PHA-L. CD4^+^ T cells were then purified as described above and re-suspended in the appropriate volume of virus in 20% FBS and 8 μg/mL polybrene. Subsequently, cells were spinoculated for 4 h at 2880×*g* at room temperature, re-suspended in fresh complete 10% RPMI and analyzed 48 h post-infection.

### Flow cytometry analysis of cell surface receptors

For cell surface staining of Sup-T1 cells, 48 h post infection, cells were washed twice with PBS, followed by staining for 20 min at room temperature with 1:6000 Zombie Red^TM^ (BioLegend) in PBS, where appropriate. Cells were then washed in FACS buffer (1% FBS, 50 mM ethylenediaminetetraacetic acid (EDTA) in PBS) and fixed in 1% paraformaldehyde (PFA) for 20 min at room temperature. Cells were subsequently washed twice with FACS buffer and stained with the appropriate antibodies by rocking for 40 min at room temperature. Cells were then washed twice with FACS buffer and re-suspended in PBS. For staining of CD28 and CD4, 1:25 APC-conjugated mouse-anti-CD28 (clone CD28.2, BioLegend) and 1:25 APC-conjugated mouse-anti-CD4 (clone OKT4, BioLegend) were utilized, respectively. For cell surface MHC-I staining, cells were stained with W6/32 (anti-MHC-I, pan-selective for HLA-A, B and C, provided by D. Johnson, Oregon Health and Science University), washed twice with FACS buffer, incubated with an APC-conjugated species specific secondary antibody, followed by washing with FACS buffer and re-suspending in PBS. For analysis of total CD28 or CD4 levels, cells were prepared as above, but were permeabilized and blocked prior to staining. Specifically, after fixation, cells were permeabilized by incubation with 0.5% saponin for 15 min at room temperature. Cells were then washed with 1% FBS, 0.1% saponin, 5 mM EDTA and blocked for 30 min with blocking buffer (10% FBS, 0.1% saponin, 5 mM EDTA in PBS). Cells were incubated with primary antibody diluted in blocking buffer followed by washing with 1% FBS, 0.1% saponin, 5 mM EDTA and re-suspending in PBS.

For cell surface staining of peripheral blood mononuclear cells infected with VSV-G pseudotyped NL4.3 encoding eGFP, cells were fixed in 2% PFA for 20 min at room temperature, followed by washing twice with cell staining buffer (BioLegend). Cells were then incubated for 40 min at room temperature with the appropriate fluorescently labeled primary antibodies (1:50 APC-Cy7 conjugated anti-CD4 (Clone OKT4, BioLegend), 1:25 APC conjugated anti-CD28 (clone CD28.2, BioLegend)). Cells were then washed twice with cell staining buffer and re-suspended in PBS prior to analysis.

For analysis of total CD28 in primary CD4^+^ T cells infected with VSV-G pseudotyped NL4.3 provirus, cells were washed twice with cell staining buffer (BioLegend) and fixed and permeabilized in BD Cytofix/Cytoperm solution (BD Biosciences, San Jose, CA). Cells were subsequently washed twice with Perm/Wash buffer (BD Biosciences) and stained for 30 min at 4 °C for CD28 level analysis [1:25 APC conjugated anti-CD28 (clone CD28.2, BioLegend)]. Cells were then washed, re-suspended in PBS and analyzed.

For analysis of cell surface CD28 on primary CD4^+^ T cells infected with replication competent NL4.3 provirus, cells were washed twice with cell staining buffer (BioLegend) and incubated for 30 min at 4 °C with fluorophore conjugated primary antibodies against the appropriate cell surface antigen [1:25 APC conjugated anti-CD28 (clone CD28.2, BioLegend)]. Cells were then washed twice with cell staining buffer and then fixed and permeabilized in BD Cytofix/Cytoperm solution (BD Biosciences, San Jose, CA). Cells were subsequently washed twice with Perm/Wash buffer (BD Biosciences) and stained for 30 min at 4 °C for p24 level analysis (1:50 anti-p24; clone KC57, Beckman Coulter). Cells were then washed, re-suspended in PBS and analyzed. The following isotype control antibodies were used in lieu of primary antibody as required: APC-Cy7 conjugated mouse IgG2b, κ isotype (clone MOPC-21, BioLegend), APC mouse IgG1 κ isotype control (clone MOPC-21, BioLegend), PE mouse IgG1κ (clone: MOPC-21, BioLegend).

Cells were analyzed using a BD Biosciences FACSCanto SORP (BD Biosciences). Data analysis was performed using FlowJo software (version 9.6.4, FlowJo LLC, Ashland, OR).

### Protein analysis

Infected Sup-T1 cells or HEK293T cells transfected using PolyJet (FroggaBio) were lysed 48 h post transduction or transfection for protein expression analysis. Briefly, cells were pelleted and lysed by rocking in lysis buffer [0.5 M HEPES, 1.25 M NaCl, 1 M MgCl_2_, 0.25 M EDTA, 0.1% Triton X-100 and 1× complete Protease Inhibitor Tablets (Roche, Indianapolis, IN)] for 1 h at 4 °C. The supernatant was then clarified by spinning at 16,100×*g* for 30 min at 4 °C, mixed with SDS-PAGE sample buffer (0.312 M Tris pH 6.8, 3.6 M 2-Mercaptoethanol, 50% glycerol, 10% SDS) and boiled at 95 °C for 10 min. Samples were run on 12 or 14% SDS-PAGE gels followed by transferring to nitrocellulose. Membranes were blocked with 5% milk in TBST (50 mM Tris, 150 mM NaCl, 0.1% Tween 20) for 1 h at room temperature, followed by incubation overnight at 4 °C with the appropriate primary antibody in 5% milk in TBST. The following primary antibodies were used: 1:1000 rabbit anti-Nef polyclonal antibody (NIH-AIDS Research and Reference Reagent program, catalog number 2949), 1:2000 rabbit anti-GFP polyclonal antibody (Clontech), 1:5000 rabbit anti-Vpu polyclonal antibody (NIH-AIDS Research and Reference Reagent program, catalog number 969), 1:2000 mouse anti-Actin monoclonal antibody (Thermo Fisher Scientific, 1:1000 rabbit anti-GAPDH polyclonal antibody (Thermo Fisher Scientific). The next day, membranes were washed three times with TBST and incubated for 2 h at room temperature with the appropriate species specific HRP-conjugated secondary antibodies (1:2000, Thermo Fisher Scientific) in 5% milk in TBST. Blots were subsequently washed and developed using ECL substrates (Millipore, Etobicoke, ON) and a C-DiGit chemiluminescence Western blot scanner (LI-COR Biosciences, Lincoln, NE).

### Microscopy

For microscopy experiments, cells were infected and treated with inhibitors as described above. Cells were then adhered to poly-l-lysine (Sigma-Aldrich) coated coverslips and fixed in 2% PFA for 10 min at room temperature. Cells were subsequently washed twice with PBS and permeabilized with methanol for 20 min. Cells were then washed twice with PBS and blocked in 2% bovine serum albumin (BSA) in PBS for 1 h at room temperature. Cells were subsequently stained for 2 h with mouse anti-CD28 (Thermo Fisher Scientific) and rabbit anti-LAMP-1 (Developmental Studies Hybridoma Bank, University of Iowa) diluted at 1:200 in 0.2% BSA in PBS, washed twice with 0.2% BSA in PBS and incubated for one and a half hours with the appropriate fluorophore conjugated secondary antibodies (Alexa-Fluor-647 conjugated anti-mouse and Cy3-conjugated anti-rabbit; Jackson ImmunoResearch, West Grove, PA) at 1:400 in 0.2% BSA in PBS. Finally, cells were washed twice with PBS and mounted on coverslips with DAPI Fluoromount-G (SouthernBiotech, Birmingham, AL). Cells were imaged on a Leica DMI6000 B at 63× or 100× magnification using the FITC, Cy3, Cy5 and DAPI filter settings and imaged with a Hamamatsu Photometrics Delta Evolve camera. Images were subsequently deconvolved using the Advanced Fluorescence Deconvolution application (Lecia, Wetzlar, Germany) on the Leica Application Suite software. Co-localization analysis was conducted using Mander’s Coefficent from the ImageJ plugin JACoP, as described previously [96].

### Cell activation analysis

To examine activation in infected cells, cryopreserved PBMCs were revived and rested for 6 h in complete RPMI media (without PHA/IL-2) at 37 ºC and 5% CO_2_. CD4^+^ T cells were then purified as described above and spinoculated with the appropriate VSV-G pseudotyped NL4.3 virus encoding both Nef and Vpu (NL4.3 dG/P eGFP), encoding Vpu alone (NL4.3 dG/P eGFP dNef), encoding Nef alone (NL4.3 dG/P eGFP dNef), or lacking both Nef and Vpu (NL4.3 dG/P eGFP dVpu dNef). After spinoculation, cells were incubated in complete 10% RPMI for 24 h prior to incubating with anti-CD3 (10 μg/ml, clone OKT3, BioLegend) immobilized on a plate and soluble anti-CD28 (5 μg/ml, clone CD28.2, BioLegend). After 24 h of activation, cells were treated with Brefeldin A (1:1000 dilution, BD Biosciences) for 12 h before proceeding for intracellular IL-2 staining.

For intracellular staining of IL-2, infected CD4^+^ T cells were fixed using Cytofix/Cytoperm solution (BD Biosciences) for 20 min at 4 °C, washed twice with Perm/Wash buffer (BD Biosciences) and stained for intracellular IL-2 with an APC-conjugated anti-IL-2 antibody (clone MQ1-17H12, BD Biosciences), or the appropriate isotype control (APC Rat IgG2a, κ isotype control, clone R35-95, BD Biosciences), for 1 h at 4 °C (1:20 dilution in Perm/Wash buffer). Cells were then washed twice using Perm/Wash buffer prior to analysis by flow cytometry. In order to determine the percentage of the infected cells that were IL-2 positive, the percentage of GFP^+^ IL-2^+^ cells (Q_2_, Fig. 8) was divided by the total percentage of infected cells (sum of GFP^+^ IL-2^+^ (Q_2_, Fig. 8) and GFP^+^ IL-2^−^ (Q_3_, Fig. 8)) and multiplied by 100.

### Data and statistical analysis

For analyses of flow cytometry data obtained for Sup-T1 cells infected with VSV-G pseudotyped NL4.3 encoding Vpu and various Nef mutations, relative levels of receptors were determined by normalizing geometric mean fluorescence intensity after gating on infected (GFP^+^) cells (Additional file 4). For all other analysis of cell surface receptors on Sup-T1 cells infected with VSV-G pseudotyped NL4.3, relative levels of receptors were determined by normalizing geometric mean fluorescence intensity after gating on single, live (Zombie Red^TM−^), infected (GFP^+^) cells (Additional file 1). Relative levels of CD28 on primary CD4^+^ T cells infected with replication competent NL4.3 were determined by normalizing geometric mean fluorescence intensity after gating on single, p24 high and CD28^+^ cells (Additional file 2). Relative levels of CD28 on VSV-G pseudotyped NL4.3 infected PBMCs, were determined by normalizing geometric mean fluorescence intensity after gating on CD4^+^ and infected (GFP^+^) lymphocytes (Additional file 7). For all cell surface or total receptor analysis by flow cytometry, the geometric mean fluorescence intensity of the control in each experiment (left-most sample on each graph) was set to 1 and the other sample MFIs were calculated relative to the control having an MFI of 1. To calculate the relative increase in intracellular staining upon ammonium chloride treatment, the geometric mean fluorescence intensity of the live and infected cells treated with ammonium chloride was divided by the geometric mean fluorescence intensity of cells that were untreated. This ratio was then normalized such that the fold increase in MFI for cells infected with NL4.3 was equal to one. All statistics for analysis of receptor levels were conducted using a one-way analysis of variance with Bonferroni’s multiple comparison test. For analysis of CD28: LAMP-1 co-localization, the mean Manders’ overlap coefficients were compared using a one-way analysis of variance with Bonferroni’s multiple comparison test to compare wild-type infected cells to cells infected with viruses encoding or lacking Nef and/or Vpu (Fig. 3) or wild-type infected cells treated with vehicle to cells treated with various inhibitors (Fig. 4). Alternatively, for analysis of the percentage of IL-2 positive infected cells, a paired two-tailed *t* test was used. All statistical tests were completed using Graph Pad Prism (Graph Pad Software Inc., La Jolla, CA).

## Additional files

**Additional file 1.** Gating of live infected Sup-T1 cells infected with Gag-Pol truncated VSV-G pseudotyped NL4.3. To examine live and infected cells, dead cells were excluded by gating on Zombie Red^TM−^ cells, doublets were excluded and subsequently infected (GFP^+^) cells were gated on. In a representative experiment, 80.3% of cells were live (Zombie Red^TM−^) and 83.1% of live single cells were infected (GFP^+^). Gates were set based on FMO (fluorescence minus one) controls stained for all fluorophores except that which is being gated on.

**Additional file 2.** Analysis of primary CD4^+^ T cells infected with replication competent virus. Primary CD4^+^ T cells were purified and infected with replication competent NL4.3 viruses and stained for cell surface CD28 and intracellular p24. (A) To examine the cells surface CD28 levels on infected cells, single cells were gated on followed by gating on the p24 (PE) and CD28 (APC) high population. In a representative experiment, 5.22% of cells were p24 high. (B) Representative dot plots illustrating p24 (PE) and CD28 (APC) on the following groups: uninfected and unstained, stained with the appropriate APC isotype control, infected and stained with the appropriate PE isotype control, or infected with the indicated viruses and stained with both anti-CD28 (APC) and anti-p24 (PE).

**Additional file 3.** Ammonium chloride treatment increases total CD4 levels in infected cells. CD4^+^ Sup-T1 cells were infected with Gag-Pol truncated VSV-G pseudotyped NL4.3 encoding or lacking Nef and/or Vpu. Infected cells were treated with 40 mM ammonium chloride for 48 h prior to staining for CD4 and analyzed by flow cytometry. (A) Representative histograms illustrating CD4 (APC) levels on live, infected cells. Mean geometric fluorescence intensities (MFIs) are indicated. (B) MFIs of infected cells were determined after gating on live, infected (Zombie Red^TM−^ and GFP^+^) cells and the relative fold increase (± SE) in total CD4 (n ≥ 5) upon ammonium chloride treatment is illustrated. (SE: standard error; ****p ≤ 0.0001).

**Additional file 4.** Gating of Sup-T1 cells infected with VSV-G pseudotyped NL4.3 encoding various Nef mutants. To examine the population of interest, cells were gated on, followed by gating on infected (GFP^+^) cells. In a representative experiment 97.9% of cells were infected (GFP^+^).

**Additional file 5.** Nef: host protein interaction motifs are critical for Nef-mediated CD28 downregulation in the presence of Vpu. Infected CD4^+^ Sup-T1 cells were stained for CD28 or MHC-I and analyzed by flow cytometry. Cells infected with VSV-G pseudotyped wild-type NL4.3 (NL4.3, red) or NL4.3 lacking Nef (dNef, blue) were used as controls. (A) Mean (± SE) relative cell surface CD28 of cells infected with NL4.3 encoding various mutations in the *nef* gene (n ≥ 5). (B) Mean (± SE) relative cell surface MHC-I on cells infected with NL4.3 encoding various *nef* mutations (n ≥ 4). (C) Relative mean (± SE) total CD28 within live cells infected with NL4.3 encoding various *nef* mutations (n ≥ 6). (SE: standard error; *p ≤ 0.05; **p ≤ 0.01; ****p ≤ 0.0001).

**Additional file 6.** Specific motifs in Vpu are critical for downregulation of CD4. Infected CD4^+^ Sup-T1 cells were stained for CD4 and analyzed by flow cytometry. Mean geometric fluorescence intensities of cells (MFI) were determined after gating on live and infected (Zombie Red^TM−^ and GFP^+^) cells. Cells infected with VSV-G pseudotyped NL4.3 lacking Nef (dNef, blue) and both Nef and Vpu (dNef dVpu, green) were used as controls. (A) Mean (± SE) relative cell surface CD4 on cells infected with NL4.3 encoding various mutations *in vpu* (n ≥ 4). (B) Relative mean (± SE) total CD4 within cells infected with NL4.3 encoding various Vpu mutations (n ≥ 5). (SE: standard error; *p ≤ 0.05; **p ≤ 0.01; ***p ≤ 0.001; ****p ≤ 0.0001).

**Additional file 7.** Gating of CD4^+^ peripheral blood mononuclear cells infected with VSV-G pseudotyped NL4.3. To examine the population of interest, lymphocytes were gated on, followed by gating on CD4^+^ (APC-Cy7) positive and infected (GFP^+^) cells. In a representative experiment 35.9% of lymphocytes were CD4^+^ and 1.3% of these were infected (GFP^+^). Gates were set based on isotype stained (APC-Cy7) and uninfected controls.

## Abbreviations

CTLs: cytotoxic T lymphocytes
APCs: antigen presenting cells
TCRs: T cell receptors
HIV-1: human immunodeficiency virus type-I
HTLV: human T-lymphotropic virus
EBV: Epstein–Barr virus
AP-1: adaptor protein 1
AP-2: adaptor protein 2
MHC-I: major histocompatibility complex class I
BST-2: bone marrow stromal antigen 2
ERAD: endoplasmic-reticulum-associated protein degradation
PBMC: peripheral blood mononuclear cells
LAMP-1: lysosomal-associated membrane protein 1
DMSO: dimethyl sulfoxide
CTLA-4: cytotoxic T-lymphocyte-associated protein 4
PAK2: p21 activated kinase 2
NF- κB: nuclear factor kappa-light-chain-enhancer of activated B cells
DMEM: Dulbecco’s modified Eagle’s medium
RPMI: Roswell Park Memorial Institute Media
PHA: phytohemagglutinin
PBS: phosphate buffered saline
FBS: fetal bovine serum
EDTA: ethylenediaminetetraacetic acid
PFA: paraformaldehyde
