# Supplementary figures and images for: The HIV-1 accessory proteins Nef and Vpu downregulate total and cell surface CD28 in CD4+ T cells
Source: Retrovirology. 2018 Jan 12;15:6. doi: 10.1186/s12977-018-0388-3 (PMC5767034; doi:10.1186/s12977-018-0388-3)

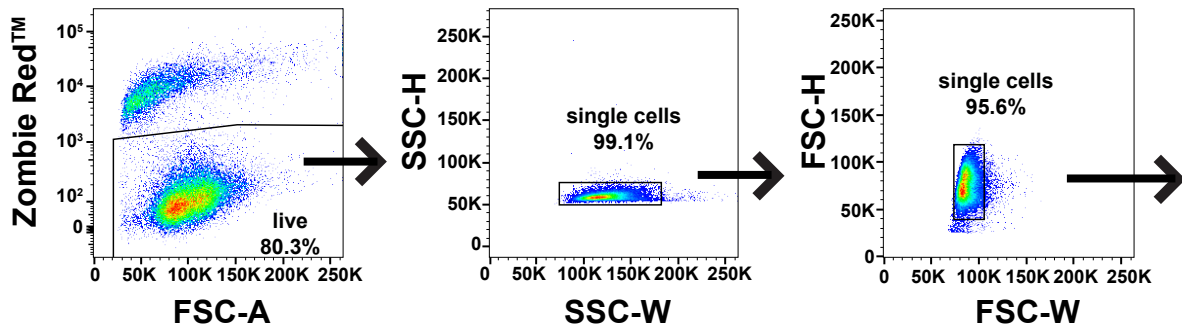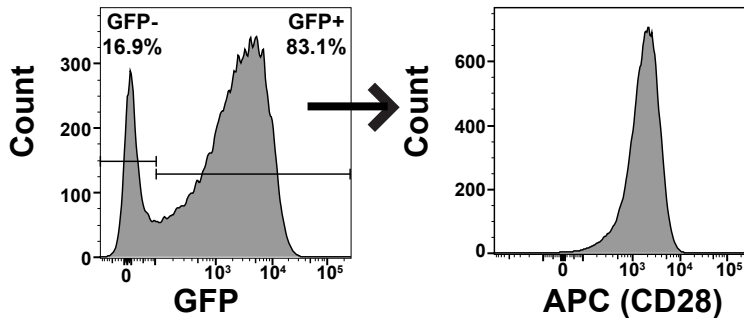

Supplement: Supplementary file 1 — Additional file 1. Gating of live infected Sup-T1 cells infected with Gag-Pol truncated VSV-G pseudotyped NL4.3. To examine live and infected cells, dead cells were excluded by gating on Zombie RedTM− cells, doublets were excluded and subsequently infected (GFP+) cells were gated on. In a representative experiment, 80.3% of cells were live (Zombie RedTM−) and 83.1% of live single cells were infected (GFP+). Gates were set based on FMO (fluorescence minus one) controls stained for all fluorophores except that which is being gated on. [file 12977_2018_388_MOESM1_ESM.pdf]

**A.**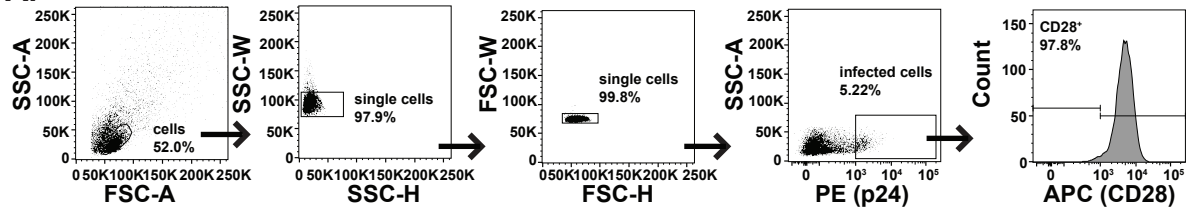**B.**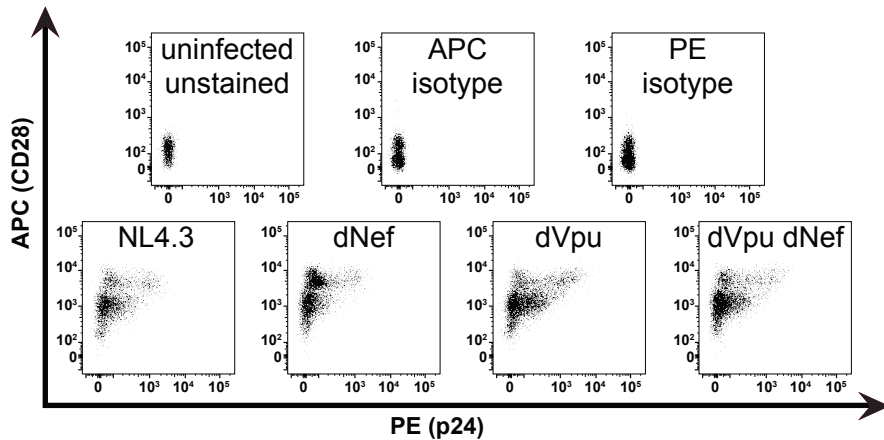

Supplement: Supplementary file 2 — Additional file 2. Analysis of primary CD4+ T cells infected with replication competent virus. Primary CD4+ T cells were purified and infected with replication competent NL4.3 viruses and stained for cell surface CD28 and intracellular p24. (A) To examine the cells surface CD28 levels on infected cells, single cells were gated on followed by gating on the p24 (PE) and CD28 (APC) high population. In a representative experiment, 5.22% of cells were p24 high. (B) Representative dot plots illustrating p24 (PE) and CD28 (APC) on the following groups: uninfected and unstained, stained with the appropriate APC isotype control, infected and stained with the appropriate PE isotype control, or infected with the indicated viruses and stained with both anti-CD28 (APC) and anti-p24 (PE). [file 12977_2018_388_MOESM2_ESM.pdf]

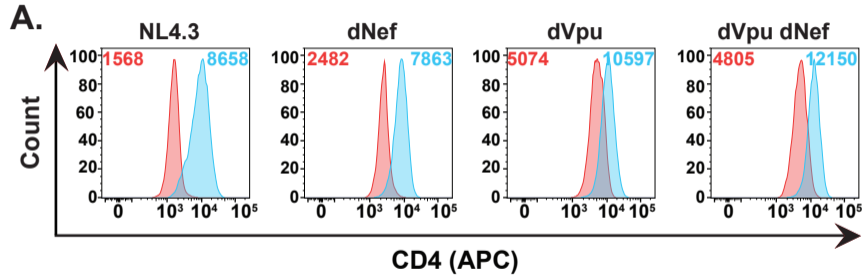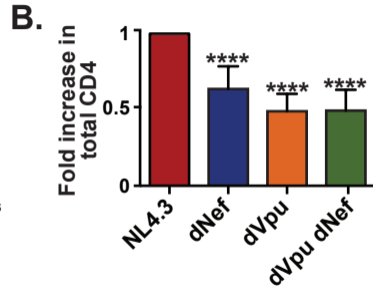

Supplement: Supplementary file 3 — Additional file 3. Ammonium chloride treatment increases total CD4 levels in infected cells. CD4+ Sup-T1 cells were infected with Gag-Pol truncated VSV-G pseudotyped NL4.3 encoding or lacking Nef and/or Vpu. Infected cells were treated with 40 mM ammonium chloride for 48 h prior to staining for CD4 and analyzed by flow cytometry. (A) Representative histograms illustrating CD4 (APC) levels on live, infected cells. Mean geometric fluorescence intensities (MFIs) are indicated. (B) MFIs of infected cells were determined after gating on live, infected (Zombie RedTM− and GFP+) cells and the relative fold increase (± SE) in total CD4 (n ≥ 5) upon ammonium chloride treatment is illustrated. (SE: standard error; ****p ≤ 0.0001). [file 12977_2018_388_MOESM3_ESM.pdf]

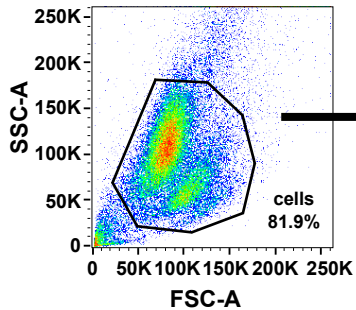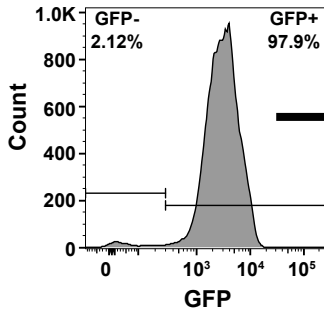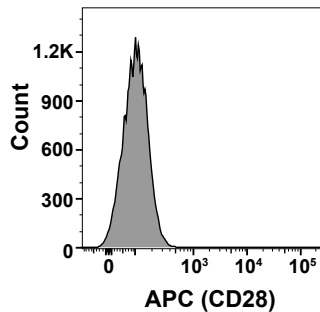

Supplement: Supplementary file 4 — Additional file 4. Gating of Sup-T1 cells infected with VSV-G pseudotyped NL4.3 encoding various Nef mutants. To examine the population of interest, cells were gated on, followed by gating on infected (GFP+) cells. In a representative experiment 97.9% of cells were infected (GFP+). [file 12977_2018_388_MOESM4_ESM.pdf]

A.

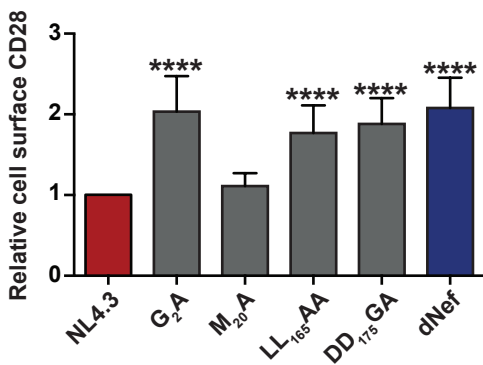

B.

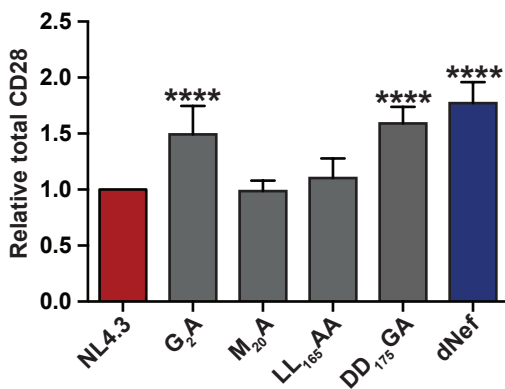

C.

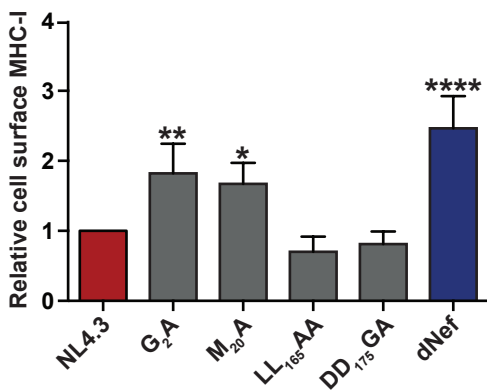

Supplement: Supplementary file 5 — Additional file 5. Nef: host protein interaction motifs are critical for Nef-mediated CD28 downregulation in the presence of Vpu. Infected CD4+ Sup-T1 cells were stained for CD28 or MHC-I and analyzed by flow cytometry. Cells infected with VSV-G pseudotyped wild-type NL4.3 (NL4.3, red) or NL4.3 lacking Nef (dNef, blue) were used as controls. (A) Mean (± SE) relative cell surface CD28 of cells infected with NL4.3 encoding various mutations in the nef gene (n ≥ 5). (B) Mean (± SE) relative cell surface MHC-I on cells infected with NL4.3 encoding various nef mutations (n ≥ 4). (C) Relative mean (± SE) total CD28 within live cells infected with NL4.3 encoding various nef mutations (n ≥ 6). (SE: standard error; *p ≤ 0.05; **p ≤ 0.01; ****p ≤ 0.0001). [file 12977_2018_388_MOESM5_ESM.pdf]

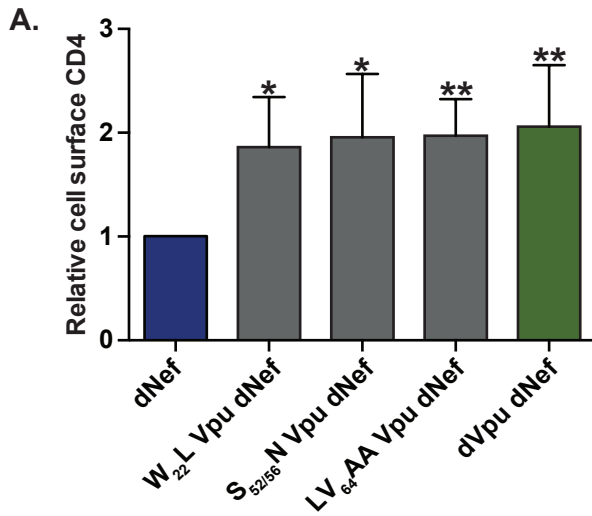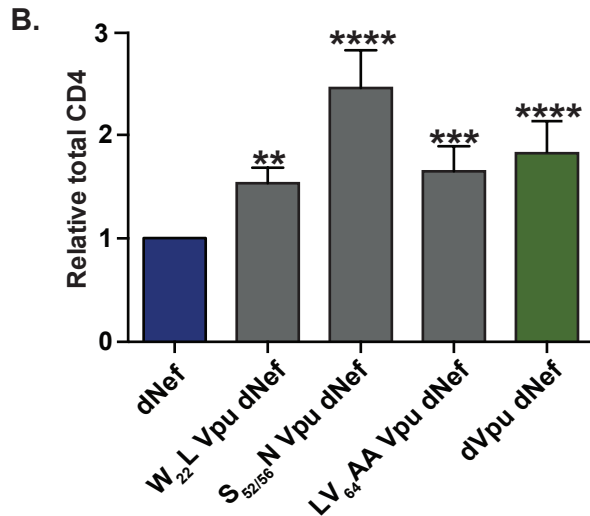

Supplement: Supplementary file 6 — Additional file 6. Specific motifs in Vpu are critical for downregulation of CD4. Infected CD4+ Sup-T1 cells were stained for CD4 and analyzed by flow cytometry. Mean geometric fluorescence intensities of cells (MFI) were determined after gating on live and infected (Zombie RedTM− and GFP+) cells. Cells infected with VSV-G pseudotyped NL4.3 lacking Nef (dNef, blue) and both Nef and Vpu (dNef dVpu, green) were used as controls. (A) Mean (± SE) relative cell surface CD4 on cells infected with NL4.3 encoding various mutations in vpu (n ≥ 4). (B) Relative mean (± SE) total CD4 within cells infected with NL4.3 encoding various Vpu mutations (n ≥ 5). (SE: standard error; *p ≤ 0.05; **p ≤ 0.01; ***p ≤ 0.001; ****p ≤ 0.0001). [file 12977_2018_388_MOESM6_ESM.pdf]

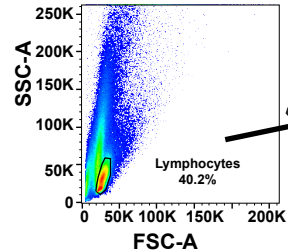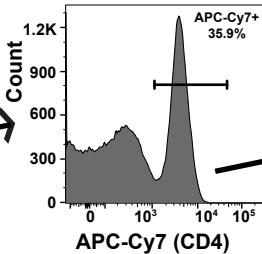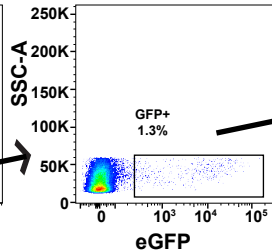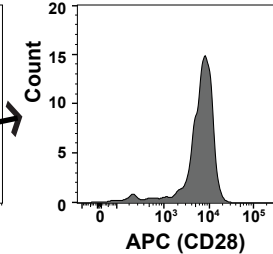

Supplement: Supplementary file 7 — Additional file 7. Gating of CD4+ peripheral blood mononuclear cells infected with VSV-G pseudotyped NL4.3. To examine the population of interest, lymphocytes were gated on, followed by gating on CD4+ (APC-Cy7) positive and infected (GFP+) cells. In a representative experiment 35.9% of lymphocytes were CD4+ and 1.3% of these were infected (GFP+). Gates were set based on isotype stained (APC-Cy7) and uninfected controls. [file 12977_2018_388_MOESM7_ESM.pdf]
